# Ancient Whole-Genome Duplication and Lineage-Specific Retention Shape the Diversification of bZIP Transcription Factors in Pooideae

**DOI:** 10.3390/plants15111608

**Published:** 2026-05-23

**Authors:** Xiaoxue Xie, Jiapeng Han, Huazhen Xu, Yuesheng Wang, Mingjie Chen, Junli Chang, Yin Li, Guangxiao Yang, Guangyuan He

**Affiliations:** 1 The Genetic Engineering International Cooperation Base of Chinese Ministry of Science and Technology, Key Laboratory of Molecular Biophysics of Chinese Ministry of Education, College of Life Science and Technology, Huazhong University of Science and Technology, Wuhan 430074, Chinayinli2021@hust.edu.cn (Y.L.); 2 State Key Laboratory of Plant Diversity and Specialty Crops, Wuhan Botanical Garden, Chinese Academy of Sciences, Wuhan 430074, China; hanjiapeng@wbgcas.cn; 3 Laboratory Equipment and Management Office, Nanjing University, Nanjing 210023, China

**Keywords:** bZIP, evolution, Pooideae, gene duplication, gene expression

## Abstract

Gene duplication is a primary evolutionary driver of gene family expansion and functional diversification in plants, yet how different duplication processes reshape the evolutionary architecture of transcription factor repertoires remains poorly resolved in lineage-specific genomic contexts. Here, we performed a comprehensive evolutionary and transcriptomic analysis of the basic leucine zipper (*bZIP*) family across 17 representative species, with a focus on Pooideae. We identified 1878 *bZIP* genes and found that, although copy numbers were relatively conserved in most diploid grasses, polyploid Triticeae showed substantial expansion. Genome-wide and *Ks* analyses indicated that *bZIP* genes were preferentially retained after whole-genome/segmental duplication, with many copies tracing back to the ancient grass-specific ρ-WGD event, the most recent shared polyploidization event in Poaceae. Phylogenetic analyses and orthology inference further resolved four evolutionary models linking ancient duplication with lineage-specific retention and expansion. Transcriptome analyses revealed structured expression divergence across developmental and stress-related contexts, and wheat homoeologous triads exhibited widespread subgenome expression bias that was dynamically reconfigured under stress and hormone treatments. Differences in transposable element landscapes among duplication models and subgenomes further suggest a role for local genomic context in regulatory divergence. Together, these findings establish a unified framework linking ancient duplication, selective retention, and transcriptional diversification of the *bZIP* family in Pooideae.

## 1. Introduction

Gene duplication is a major force driving gene family expansion and functional diversification during plant genome evolution [1]. Duplicated genes provide the raw genetic material for evolutionary innovation and may be retained, lost, or functionally differentiated through changes in coding sequence or gene regulation [2]. In plants, both whole-genome duplication (WGD) and small-scale duplications (SSDs), such as tandem duplication (TD) and dispersed duplications (DD), contribute to gene family evolution; however, their evolutionary consequences are often unequal. WGD/segmental duplicates are typically retained from large-scale chromosomal or whole-genome duplication events, whereas TD genes occur as adjacent duplicated copies, proximal duplication (PD) genes are duplicated loci separated by only a few intervening genes, and DD genes are duplicated copies distributed at distant genomic locations. In contrast, singleton genes lack detectable duplicated counterparts within the genome [3,4,5,6]. In particular, transcription factor families are often preferentially retained following WGD, likely because their functions within dosage-sensitive regulatory networks favor the maintenance of duplicated copies, suggesting that duplication history can have long-lasting effects on the composition and functional diversification of regulatory gene repertoires [7,8]. Although genome-wide patterns of gene duplication have been extensively characterized in many plant lineages [2,9,10], how different duplication modes and subsequent lineage-specific retention jointly shape the evolution of individual transcription factor families remains poorly understood.

The basic leucine zipper (bZIP) transcription factor family is one of the largest and most conserved transcription factor families in plants and is characterized by a highly conserved bZIP domain [11,12]. The bZIP domain consists of an invariant N-x7-R/K motif responsible for recognizing ACGT-core *cis*-elements (e.g., G-boxes/ABREs) and a leucine zipper region that mediates homo- or heterodimer formation through a characteristic heptad repeat of hydrophobic residues [13,14]. Plant *bZIP* transcription factors play essential roles in a wide range of biological processes, including seed development, hormone signaling, abiotic stress responses, and metabolic regulation [13,14]. *bZIP* genes are typically classified into twelve deeply conserved groups (i.e., A, B, C, D, E, F, G, H, I, J, K, and S) [15,16]. These groups exhibit considerable divergence in sequence architecture, DNA-binding specificity, and physiological roles [14,15,17,18]. For example, group A members are primarily associated with ABA signaling and abiotic stress responses [19]; groups B and K participate in ER stress and unfolded protein response pathways [20]; groups C and S regulate energy homeostasis and nutrient signaling [21]; group D (TGA factors) functions in pathogen defense and salicylic acid signaling [22]; group E is linked to reproductive development [23]; group F regulates zinc deficiency and ion homeostasis [24]; groups G and H are involved in light signaling and photomorphogenesis [25,26]; group I participates in developmental and stress signaling pathways [27]; whereas groups J and K represent relatively small and structurally divergent lineages with specialized functions [14]. Because of their central roles in development and stress adaptation, *bZIP* genes provide an excellent model for investigating how gene duplication contributes not only to gene family expansion but also to regulatory diversification. Although some evolutionary features identified in this study may also apply to other transcription factor families, the comparatively conserved structure and classification of *bZIPs* facilitates integrated analyses of duplication history, retention, and transcriptional divergence across grasses. Previous studies have identified and characterized *bZIP* genes in *Arabidopsis thaliana*, *Oryza sativa*, *Hordeum vulgare*, *Triticum aestivum*, and several other species, providing insights into the functional roles and phylogenetic relationships of different genes [14,18,28,29]. However, most of these studies have focused on single-species analyses, and comparative evolutionary investigations of the *bZIP* family across grasses remain limited.

Pooideae represents one of the most economically and ecologically important grass lineages, encompassing important temperate cereals such as wheat and barley [30,31]. Pooideae has a complex evolutionary history involving ancient whole-genome duplication, lineage-specific retention and loss, and recent polyploidization events [3,30,31,32]. In particular, the grass-specific ancient ρ-WGD event and the recent burst of gene duplications are thought to have had a lasting impact on gene retention across Pooideae [3,6]. The coexistence of diploid and polyploid lineages within this subfamily provides a valuable comparative framework for investigating how duplication history and subsequent evolutionary processes shape transcription factor families such as bZIP. Nevertheless, systematic identification and evolutionary analyses of *bZIP* genes have been limited to a small number of species, including *Brachypodium distachyon*, *H. vulgare*, *T. aestivum*, *Triticum Urartu*, and *Aegilops tauschii* [18,29,33,34], leaving the evolutionary dynamics of this gene family across Pooideae largely unresolved.

Beyond gene retention, transcriptional divergence is a critical component of functional diversification after gene duplication [35]. Duplicated genes often exhibit changes in expression level, spatial–temporal pattern, or responsiveness to environmental stimuli, which can facilitate both sub- and neofunctionalization [36,37,38,39]. In polyploid species, this process is further shaped by the presence of multiple subgenomes [32,40]. Homoeologous genes derived from different subgenomes frequently show unequal expression contributions, a phenomenon referred to as subgenome expression bias [36]. This bias can be stable across developmental contexts or dynamically reconfigured under environmental conditions, reflecting both inherited expressional differences and ongoing regulatory divergence [41]. However, the extent to which duplication history influences expression divergence and subgenome partitioning, particularly in transcription factor families such as bZIP, remains poorly understood.

In this study, we conducted a comprehensive identification and evolutionary analysis of *bZIP* genes across 17 representative species. We aimed to address three key questions: (i) Which duplication mechanisms underlie the expansion of the *bZIP* gene family across grasses? (ii) How do ancient duplication and lineage-specific retention jointly shape the diversification of *bZIP* genes in Pooideae? and (iii) How are duplicated *bZIP* genes transcriptionally partitioned during development and under stress conditions, particularly in polyploid wheat? By integrating phylogenetic reconstruction, duplication type classification, synteny analysis, transcriptome profiling, and transposable element (TE) analyses, our results provide a unified framework linking duplication history, gene retention, and transcriptional divergence in *bZIP* family evolution. Our analyses revealed that WGD/segmental duplication constitutes the predominant source of retained *bZIP* genes across Pooideae and identified four distinct evolutionary models linking ancient duplication with lineage-specific diversification. We further found widespread transcriptional divergence and dynamic subgenome expression bias among wheat *bZIP* homoeologs, suggesting that both duplication history and post-polyploid divergence contribute to functional diversification of the family.

## 2. Results

### 2.1. Identification and Copy Number Variation in bZIP Genes Across Pooideae

**Figure 1:**
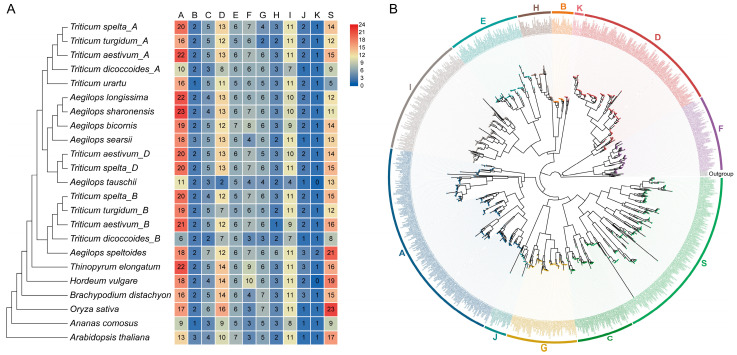
Identification and phylogenetic classification of *bZIP* genes across grasses. (**A**) Copy number variation and subfamily composition of *bZIP* genes across 17 representative species. The phylogenetic relationships among species are shown on the left. The heatmap displays the number of *bZIP* genes in each of the 12 subfamilies (A–K and S) for each species. Color intensity represents gene counts, with red indicating higher numbers and blue indicating lower numbers. (**B**) Maximum likelihood phylogenetic tree of bZIP proteins constructed from all identified sequences. Genes are grouped into 12 major subfamilies (A–K and S), indicated by colored sectors along the outer ring. HD-ZIP sequences from *A. thaliana* were included as outgroup. Branches are colored according to group classification.

To investigate the evolutionary expansion of the *bZIP* family in grasses, we performed a comprehensive identification of bZIP domain-containing proteins across 17 representative species, encompassing 14 Pooideae species, *O. sativa*, *Ananas comosus*, and *A. thaliana*. Among these, nine species were systematically analyzed for the first time, including four *Aegilops* species (*Aegilops bicornis*, *Aegilops longissima*, *Aegilops searsii*, and *Aegilops sharonensis*), three *Triticeae* species (*Triticum dicoccoides*, *Triticum spelta*, and *Triticum turgidum*), *Thinopyrum elongatum*, and *A. comosus*. In total, 1878 *bZIP* genes were identified (Figure 1A and Table S1). Across diploid grasses, including *O. sativa* and non-Triticeae Pooideae species, the size of the *bZIP* family was relatively conserved. For example, *O. sativa*, *B. distachyon*, and *H. vulgare* contained 96, 87, and 94 *bZIP* genes, respectively. This overall consistency suggests that the ancestral grass genome likely maintained a relatively stable complement of *bZIP* genes. In contrast, among the *Aegilops* species, considerable variation in gene number was observed despite their diploid genome status, with 51–101 *bZIP* genes detected in each species. This variation indicates that lineage-specific gene retention and loss have contributed to differences in family size.

Polyploid Triticeae species exhibited much greater expansion, with *bZIP* copy numbers ranging from 110 to 269, highlighting the major contribution of polyploidization to the expansion of the *bZIP* family. Genome assembly quality also influences gene family identification. For instance, a previous study reported 187 *bZIP* genes in the hexaploid bread wheat [18], whereas our analysis based on the recently released telomere-to-telomere (T2T) wheat genome assembly identified 269 genes. The increase of 82 newly identified wheat *bZIP* genes compared with previous studies is likely attributable to the improved completeness and continuity of the T2T genome assembly and annotation.

On the basis of homologous sequence alignments and evolutionary relationships with well-characterized bZIP proteins from Arabidopsis and rice, our phylogenetic analyses of grass bZIP proteins recovered twelve well-supported groups (i.e., A–K and S) (Table S2) consistent with previously reported monocot classification frameworks [13,14]. To further resolve the evolutionary relationships of these genes, we constructed a maximum likelihood (ML) phylogenetic tree using representative bZIP sequences, with Arabidopsis HD-ZIP proteins as outgroups. The results showed that all *bZIP* genes consistently clustered into twelve well-supported monophyletic branches, corresponding to the twelve established gene groups (Figure 1B and Table S2). Across most diploid grass species, the distribution of genes among these groups was broadly similar and comparable to that observed in *A. thaliana*. In polyploid *Triticeae* species, however, the increase in gene number was largely proportional across groups rather than restricted to a few specific groups.

These results indicate that the overall phylogenetic framework and relative group composition of *bZIP* genes are highly conserved across grasses, whereas variation in total copy number, particularly in polyploid *Triticeae* lineage, is primarily associated with differential retention after large-scale duplication events.

### 2.2. Genome-Wide and bZIP Family-Level Duplication Landscapes Reveal Preferential Retention of bZIP Genes

**Figure 2:**
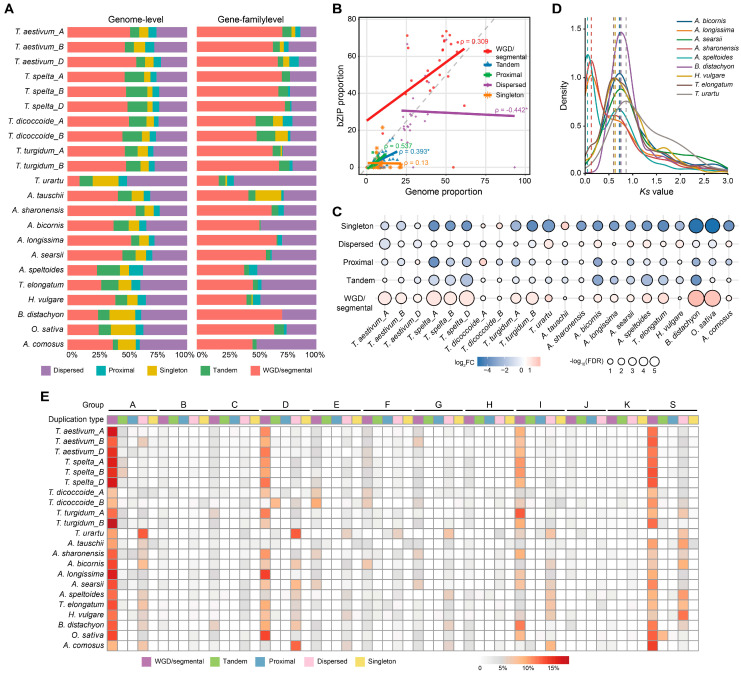
Genome-wide and family-level duplication patterns of *bZIP* genes across Poaceae. (**A**) Distribution of gene duplication types at the genome-wide and *bZIP* family levels across examined species. **Left** panels show genome-wide distributions, and **Right** panels show *bZIP*-specific distributions. (**B**) Correlation between genome-wide and *bZIP* family-level proportions of different duplication types across species. Each point represents one species, and fitted lines indicate the relationship for each duplication mode. Spearman correlation coefficients (ρ) are indicated for each regression. Statistical significance was assessed using two-tailed test, and asterisks (*) indicate *p*-value < 0.05. (**C**) Enrichment analysis of duplication types in the *bZIP* gene family relative to genome-wide expectations. Bubble size represents statistical significance (−log10 FDR), and color indicates log2 fold change. Statistical significance was assessed using hypergeometric tests with Benjamini–Hochberg correction (adjusted *p* < 0.05). (**D**) Ks distributions of syntenic gene pairs in representative grass species. Colored curves represent the Ks distributions of duplicated *bZIP* gene pairs in representative species. Dashed vertical lines indicate the major Ks peaks for each species. (**E**) Proportions of duplication types across *bZIP* groups (A–K and S) in different species. Each cell represents the percentage of genes belonging to a specific duplication mode within each subfamily.

To investigate the evolutionary mechanisms underlying the expansion of the *bZIP* gene family, we characterized gene duplication patterns across all examined species using MCScanX. Duplicated genes were classified into five categories: whole-genome/segmental duplication (WGD/segmental), tandem duplication (TD), proximal duplication (PD), dispersed duplication (DD), and singleton genes (Figure 2A and Table S3). At the genome-wide level, small-scale duplications (SSD), including DD, PD, and TD, collectively dominated the duplication landscape across species, with SSD representing the largest fraction in most genomes. WGD/segmental duplicates constituted a substantial but non-predominant proportion in most diploid species, although they were relatively more common in *Triticeae* genomes. In contrast, the *bZIP* family exhibited a distinct duplication composition. Across all examined species, WGD/segmental duplication represented the predominant source of *bZIP* genes, frequently accounting for the largest proportion of family members, whereas SSD contributed comparatively less.

To evaluate the relationship between genome-wide and family-level duplication patterns, we compared the proportion of each duplication mode across species using Spearman correlation analysis (Figure 2B). The proportion of WGD/segmental duplicates showed a positive association between genome-wide gene sets and the *bZIP* family. In contrast, DD exhibited a negative relationship, whereas TD and PD displayed weak or marginal associations. Hypergeometric enrichment analyses further confirmed this pattern (Figure 2C). WGD/segmental duplicates were significantly enriched in most examined species, whereas DD was generally depleted, and TD and PD showed no consistent enrichment pattern. In Triticeae species, WGD/segmental duplicates accounted for a large proportion of *bZIP* genes and were consistently enriched, whereas SSD contributed less prominently.

To assess whether this duplication bias also existed at the subfamily level, we compared the proportions of duplication modes across the 12 *bZIP* groups in all species (Figure 2E). Across most groups, WGD/segmental duplication and DD constituted the major components, whereas other TD and PD were less represented. Several groups (e.g., A, D, I, and S) showed relatively higher proportions of WGD/segmental duplicates than other groups. This pattern was broadly consistent across species. Thus, the duplication composition of the *bZIP* family differs from genomic background not only at the whole-family level but also among phylogenetic groups.

To further trace the evolutionary origin of retained WGD/segmental duplicates, we analyzed the synonymous substitution rate (*Ks*) distributions of syntenic *bZIP* gene pairs in representative grass species (Figure 2D). Across the examined species, *bZIP* duplicate gene pairs exhibited species-specific major Ks peaks that generally ranged from approximately 0.6 to 0.9. These major peaks were consistently observed in both Triticeae species (e.g., Aegilops spp. and *T. urartu*) and non-Triticeae grasses (e.g., *B. distachyon* and *H. vulgare*), although the exact peak positions varied slightly among lineages. In several species, including *A. longissima*, *A. sharonensis*, and *A. speltoides*, additional younger peaks (~0.05–0.15) were also detected, suggesting the occurrence of more recent lineage-specific duplication events. Importantly, the major *Ks* peak overlapped with the expected range of the Poaceae-specific ancient ρ-WGD event [3], indicating that most retained WGD/segmental-derived *bZIP* genes can be traced back to this ancestral duplication rather than to recent lineage-specific events.

Together, these results show that *bZIP* family expansion cannot be explained simply by genome-wide duplication composition. Instead, WGD/segmental duplicates are consistently enriched within the *bZIP* family across species, and the major Ks peaks of many retained duplicates overlap with the expected range of the ancient grass ρ-WGD event. These observations are consistent with a substantial contribution of ancient WGD to the diversification of the *bZIP* family in Pooideae.

### 2.3. Phylogenetic and Syntenic Analyses Reveal Four Distinct Evolutionary Models of bZIP Gene Duplication

**Figure 3:**
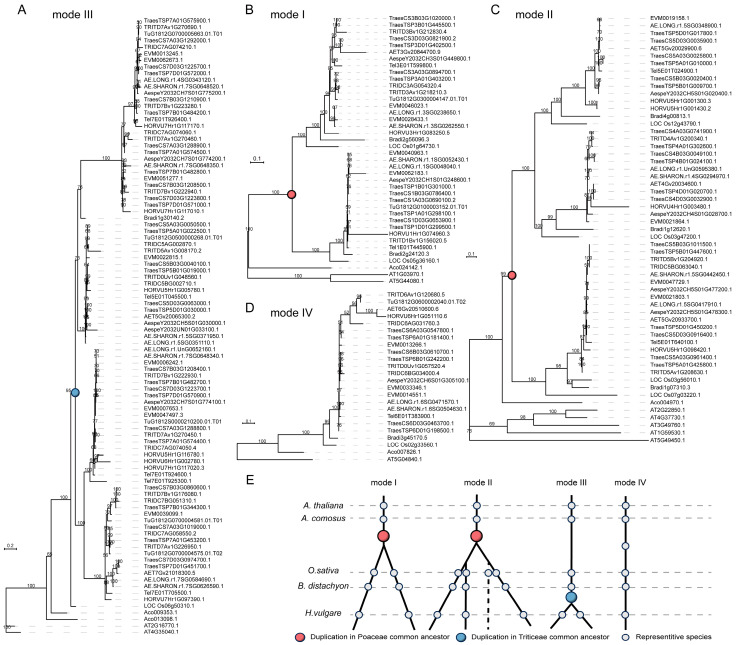
Phylogenetic reconstruction reveals four distinct evolutionary models of *bZIP* gene duplication in grasses. (**A**–**D**) Representative maximum likelihood phylogenetic trees illustrating four evolutionary models of *bZIP* gene duplication: (**A**) model III, (**B**) model I, (**C**) model II, (**D**) model IV. Each tree corresponds to a representative clade defined based on *Ananas comosus* as the reference node. Colored circles indicate inferred duplication events, with red representing duplication in the common ancestor of Poaceae and blue indicating duplication in the common ancestor of Triticeae. Bootstrap support values are shown at major nodes. (**E**) Schematic representation summarizing the four duplication models. Horizontal dashed lines represent representative species at key evolutionary nodes.

To further investigate whether different *bZIP* lineages followed similar or distinct evolutionary trajectories after duplication, we further defined 46 monophyletic clades from the twelve major groups using *A. comosus* as the reference node and classified them into four representative evolutionary models based on the phylogenetic positions of duplication events (Figure 3E and Figure S1 and Table S2). For each model, we selected representative clades and examined their phylogenetic relationships. In model I, duplication events were inferred to have occurred in the common ancestor of Poaceae. Phylogenetic trees showed well-supported duplication nodes predating species divergence, with duplicated gene lineages retained across nearly all examined species (Figure 3A,B). These clades therefore represent anciently duplicated lineages that were broadly preserved during grass evolution. Model II included clades in which an ancestral duplication was followed by additional lineage-specific expansion and differential retention. In these clades, phylogenetic analyses revealed multiple descendant subclades derived from an ancestral duplication node, indicating that ancestral duplicates remained evolutionarily active and subsequently diversified in particular lineages (Figure 3C). In model III, duplication events were largely restricted to specific lineages, especially *Triticeae*. Phylogenetic trees showed lineage-specific clustering of duplicated genes, consistent with more recent expansion after divergence from other grasses (Figure 3A). These clades likely represent lineage-restricted duplication histories that contributed to local expansion of the *bZIP* repertoire. In contrast, model IV included clades that generally retained a single gene copy per species and showed no clear evidence of retained duplication (Figure 3D). These genes therefore appear to have remained largely single-copy across grass evolution, suggesting stronger constraints on duplicate retention.

These results indicate that *bZIP* family diversification in Pooideae does not follow a single pattern. Instead, different clades reflect distinct combinations of ancient duplication, lineage-specific retention, expansion, and loss, revealing multiple evolutionary routes by which the family was shaped.

### 2.4. Expression Divergence Among Duplicated bZIP Genes Across Developmental and Stress Conditions

**Figure 4:**
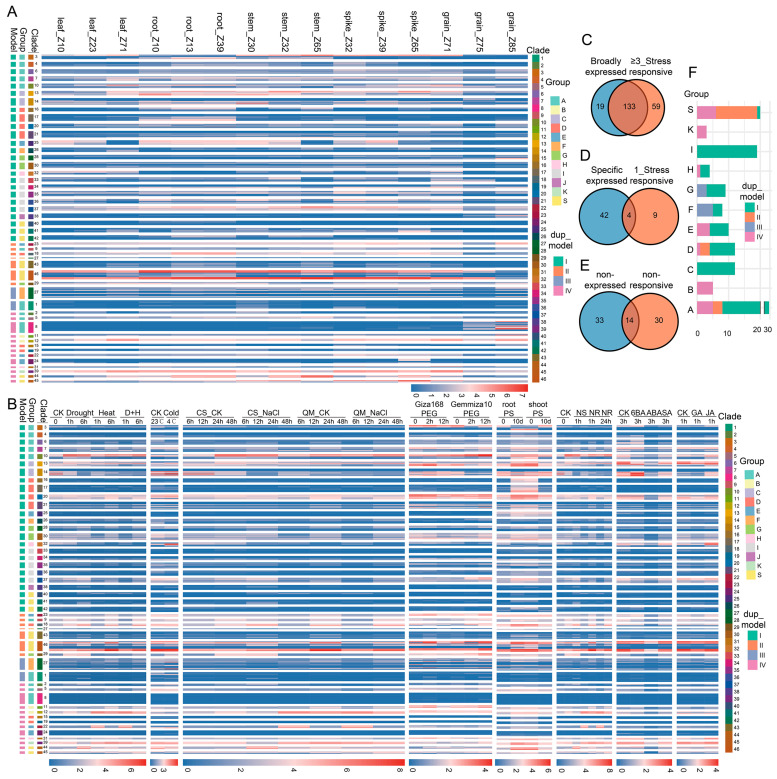
Expression divergence of *bZIP* genes across developmental stages and stress/hormone conditions in wheat. (**A**) Expression profiles of *bZIP* genes across multiple tissues and developmental stages. Heatmap shows normalized expression levels (log_2_(TPM+1)) across leaf, root, stem, spike, and grain tissues. Genes are ordered according to phylogenetic clades, with annotations indicating duplication model, group, and clade identity. Developmental stages are defined as follows: leaf_Z10 (seedling stage), leaf_Z23 (tillering stage), leaf_Z71 (2 dpa stage); root_Z10 (seedling stage), root_Z13 (three-leaf stage), root_Z39 (flag leaf stage); stem_Z30 (1 cm spike stage), stem_Z32 (two nodes stage), stem_Z65 (anthesis stage); spike_Z32 (two nodes stage), spike_Z39 (flag leaf stage), spike_Z65 (anthesis stage); grain_Z71 (2 days post anthesis, dpa), grain_Z75 (14 dpa), and grain_Z85 (30 dpa). Color scale in heatmaps represents normalized expression levels (log_2_(TPM+1)), with blue indicating low expression and red indicating high expression. (**B**) Expression profiles of *bZIP* genes under abiotic stress and hormone treatments. Heatmap shows gene expression across multiple conditions, including drought, heat, cold, NaCl, PEG, nutrient stress (phosphate starvation (PS) and nitrogen starvation (NS)), and hormone treatments (6BA, ABA, SA, GA, JA). Time-course data are indicated for each treatment. Gene ordering and annotations are consistent with panel (**A**). (**C**–**E**) Overlap between developmental expression breadth and stress responsiveness. Numbers indicate gene counts in each category. (**F**) Distribution of broadly expressed and stress-responsive genes across *bZIP* groups and duplication models. Bar plots show the number of genes in each group (A–K, S), with colors indicating their corresponding duplication models (I–IV).

We next examined whether duplicated *bZIP* genes also differ in their expression profiles across developmental and environmental contexts. Transcriptomic analyses revealed extensive variation in expression patterns among *bZIP* genes across tissues, developmental stages, and treatment conditions (Tables S4 and S5). These differences were not randomly distributed, but instead showed clearly hierarchical organization across evolutionary levels. At the duplication model level, genes belonging to the same model shared broad expression tendencies, but substantial variation was still evident. Within each model, different phylogenetic groups showed distinct transcriptional profiles. For example, in model I, group D genes tended to show relatively higher expression in leaf tissues across multiple developmental stages, whereas group I genes maintained consistently high expression across a wider range of tissues and stages (Figure 4A). Additional divergence was observed at finer phylogenetic scales. Within model II, different clades in group S displayed clear differences in both expression intensity and expression pattern. For instance, genes in clade 46 generally showed higher expression across multiple tissues than genes in clade 43. Even within the same clade, individual paralogs frequently showed distinct expression levels, indicating that transcriptional divergence continued after clade formation.

A similar pattern was observed under stress and hormone treatments (Figure 4B). Genes within the same duplication model differed markedly in their responses across groups, clades, and individual genes. For example, in model I, genes in clade 10 (group A) were induced by heat, drought, NaCl, and nutrient stresses but were generally repressed by cold and hormone treatments, whereas genes in clade 14 (group C) showed the opposite response pattern. Within group D, some clades responded specifically to particular conditions, such as phosphate starvation, whereas others responded to multiple treatments.

Notably, genes that were broadly expressed across developmental contexts also tended to respond to multiple stress and hormone treatments. By contrast, genes with more restricted developmental expression profiles generally exhibited narrower or more condition-specific responses (Figure 4C–E). We further examined the distribution of broadly expressed and stress-responsive genes across evolutionary groups and duplication models (Figure 4F). These genes were unevenly distributed among *bZIP* groups, with higher representation in groups A, I, and S. In terms of evolutionary origin, they were predominantly derived from model I, whereas model II contributed a smaller proportion and model III genes were less frequently represented.

To determine whether the observed expression divergence was specific to polyploid wheat or also present in diploid Pooideae species, we additionally analyzed publicly available barley transcriptome data across multiple tissues and developmental stages (Figure S2). Although the barley dataset covered fewer developmental contexts than the wheat dataset, *bZIP* genes still exhibited substantial expression divergence across duplication models, groups, and clades. Genes within the same duplication model often displayed distinct tissue expression profiles, and further divergence was observed among groups and clades within individual models. In particular, model I genes generally maintained broader expression across tissues, whereas genes from models II and III more frequently exhibited restricted or clade-specific expression patterns. These observations indicate that duplication-associated transcriptional divergence is already present in a diploid Pooideae genome and is therefore not solely a consequence of recent wheat polyploidization.

Together, these results reveal widespread and hierarchically structured expression divergence among duplicated *bZIP* genes across both diploid and polyploid Pooideae species. Broadly expressed and broadly responsive genes were unevenly distributed among duplication models, groups, and clades, with model I contributing a relatively larger proportion of such genes compared with other models. In polyploid wheat, this transcriptional divergence is further accompanied by subgenome-specific expression partitioning among homoeologous triads.

### 2.5. Subgenome Expression Bias During Development Reveals Unequal Transcriptional Contribution of bZIP Homoeologs

**Figure 5:**
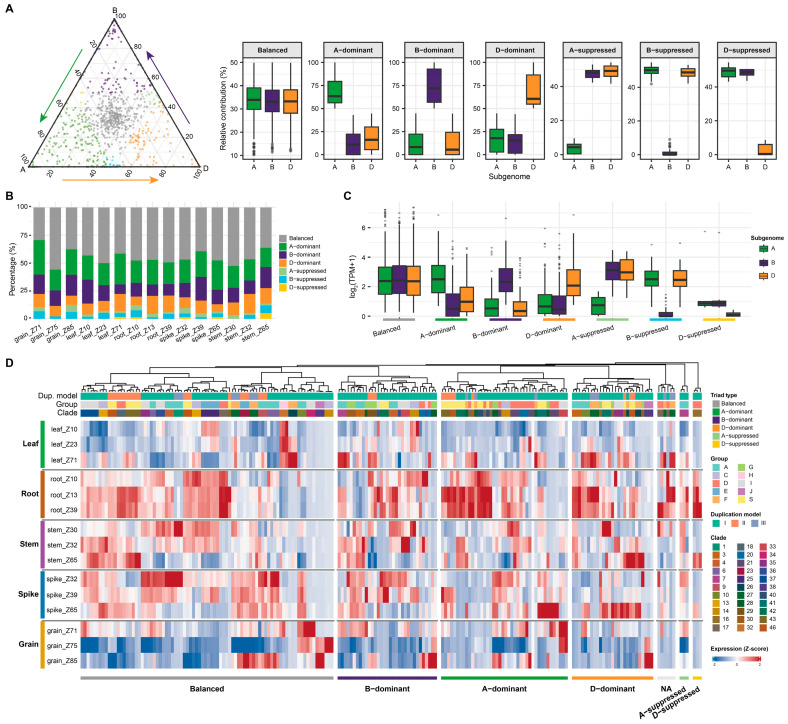
Subgenome expression bias of *bZIP* homoeologous triads across developmental stages in wheat. (**A**) Ternary plot showing the relative expression contribution of A, B, and D subgenomes for *bZIP* homoeologous triads across all tissues and developmental stages. Each point represents a triad, positioned according to the proportion of expression contributed by each subgenome. Insets display the relative expression distributions for the seven triad categories, including balanced, A/B/D-dominant, and A/B/D-suppressed types. (**B**) Proportions of triad expression categories across different tissues and developmental stages. (**C**) Expression levels of triads grouped by expression bias categories. (**D**) Heatmap showing subgenome expression bias patterns of individual *bZIP* triads across tissues and developmental stages. Each row represents a gene, and each column corresponds to a tissue-stage combination. Annotations indicate duplication model, group, clade. Color intensity represents normalized expression levels (log_2_(TPM+1). The **bottom** panel indicates triad type for each gene across samples. Developmental stages are defined as follows: leaf_Z10 (seedling stage), leaf_Z23 (tillering stage), leaf_Z71 (2 dpa stage); root_Z10 (seedling stage), root_Z13 (three-leaf stage), root_Z39 (flag leaf stage); stem_Z30 (1 cm spike stage), stem_Z32 (two nodes stage), stem_Z65 (anthesis stage); spike_Z32 (two nodes stage), spike_Z39 (flag leaf stage), spike_Z65 (anthesis stage); grain_Z71 (2 days post anthesis, dpa), grain_Z75 (14 dpa), and grain_Z85 (30 dpa).

In polyploid genomes, functional diversification of duplicated *bZIP* genes is further shaped by the coexistence of multiple subgenomes. Homoeologous genes from different subgenomes often contribute unequally to total expression, generating subgenome expression bias [42,43]. Such transcriptional divergence is thought to represent a key mechanism underlying functional partitioning following polyploidization. This raises an important question of how *bZIP* homoeologous copies are transcriptionally partitioned during plant development. To address this, we analyzed subgenome expression partitioning of *bZIP* homoeologous triads across multiple tissues and developmental stages in wheat. Triads were classified into seven categories based on the relative contributions of the A, B, and D subgenomes using the standard wheat triad partitioning framework (see Section 4), including balanced, A/B/D-dominant, and A/B/D-suppressed types (Figure 5A). At the global level, most triads were distributed near the balanced region. However, a substantial proportion showed clear subgenome expression bias, indicating unequal transcriptional contributions among homoeologs during development.

We next examined the distribution of triad categories across tissues and developmental stages (Figure 5B and Table S6). Balanced triads were consistently present, whereas other dominant categories, particularly A-, B- and D-dominant, were enriched in specific tissues and stages, suggesting that subgenome contributions are dynamically regulated during development rather than constitutive. To determine whether bias patterns were associated with expression magnitude, we compared transcript abundance among triad categories (Figure 5C). Dominant triads generally exhibited elevated expression levels in the corresponding dominant subgenome, whereas suppressed triads showed markedly reduced expression. Thus, developmental subgenome bias reflects both differences in relative contribution and divergence in absolute transcript level.

We further examined the relationship between subgenome expression bias and duplication history (Figure 5D). Triads derived from duplication model I exhibited the highest frequency of expression bias (57.6%), compared with models II (31.5%) and III (26.2%) (Fisher’s exact test, *p* = 2.59 × 10^−9^). Group-level analyses further revealed non-random distributions of biased model I triads across phylogenetic groups, with significant enrichment detected in groups S and F, whereas groups C, D, G, and H showed lower-than-expected frequencies. In contrast, only a minority of model II (approximately 4 out of 11) exhibited biased expression, while model III bias displayed comparatively limited and group-restricted expression bias patterns. Importantly, duplication origin alone could not fully explain subgenome expression partitioning. Even within the same duplication model, phylogenetic group, and clade, different triads frequently exhibited distinct expression categories across tissues and treatments. These findings suggest that although evolutionary origin influences the propensity for subgenome expression bias, the specific transcriptional partitioning patterns are further shaped by regulatory divergence following wheat polyploidization.

### 2.6. Stress and Hormone Treatments Induce Dynamic Reconfiguration of Subgenome Expression Bias in bZIP Homoeologous Triads

**Figure 6:**
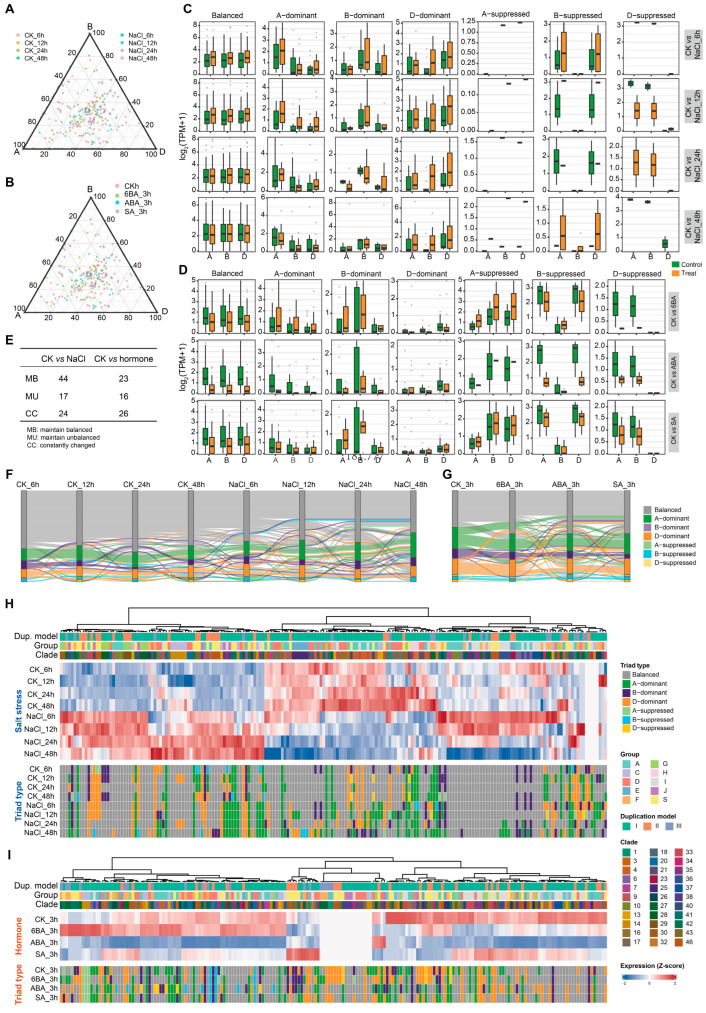
Dynamic reconfiguration of subgenome expression bias in *bZIP* homoeologous triads under stress and hormone treatments. (**A**,**B**) Ternary plots showing the relative expression contributions of A, B, and D subgenomes for *bZIP* triads under (**A**) NaCl stress and (**B**) 6BA, ABA, and SA treatments. Each point represents a triad, positioned according to the proportion of expression derived from each subgenome at different time points or treatment conditions. Colors indicate different treatments. (**C**,**D**) Expression levels of triads grouped by bias categories under (**C**) NaCl stress and (**D**) hormone treatments. Boxplots show normalized expression levels (log_2_(TPM+1)) for each subgenome within different triad types (balanced, A/B/D-dominant, and A/B/D-suppressed), comparing control and treatment conditions across time points. (**E**) Summary of triad classification dynamics under NaCl and hormone treatments. Triads were categorized into three groups based on changes in expression bias among treatment conditions: maintain balanced (MB), maintain unbalanced (MU), and constantly changed (CC). (**F**,**G**) Sankey plots illustrating dynamic transitions of triad types across (**F**) time points under NaCl stress and (**G**) across hormone treatments. (**H**,**I**) Heatmap showing subgenome expression bias patterns of individual *bZIP* triads under (**H**) NaCl stress and (**I**) hormone treatments. **Upper** panels show normalized expression levels (z-score transformed log_2_(TPM+1)) across treatments, with annotations indicating duplication model, phylogenetic group, and clade. **Lower** panels show triad classification states for each triad under corresponding conditions. Triads are hierarchically clustered based on expression profiles.

Although subgenome expression partitioning of *bZIP* homoeologous triads is established during development, it remained unclear whether this bias is maintained or reconfigured under environmental stimuli. To address this, we analyzed triad expression partitioning under NaCl treatments at multiple time points (6, 12, 24, and 48 h) and under representative hormone treatments (3 h 6-BA, ABA, and SA). Based on relative expression contributions from the A, B, and D subgenomes, triads were again classified into the same seven categories described above. Ternary plots were used to compare the overall distribution of triads before and after treatments (Figure 6A,B and Table S7). Under both NaCl and hormone treatments, triads exhibited noticeable shifts in their distribution in the ternary space, although the overall distribution remained relatively concentrated, suggesting that subgenome expression bias is modulated by external stimuli but is not completely reorganized.

We next quantified the proportion of different triad categories before and after treatments (Figure 6C,D). Under NaCl treatment, the proportion of balanced triads remained relatively stable, whereas dominant and suppressed types changed to varying degrees. In contrast, under hormone treatments, all triad categories showed more pronounced shifts, indicating that hormone signals exert a broader effect on subgenome expression partitioning than NaCl stress.

To characterize triad dynamics more explicitly, triads were further grouped into three categories according to their behavior across conditions: maintain balanced (MB), maintain unbalanced (MU), and constantly changed (CC) (Figure 6E–G). The relative abundance of these categories varied among treatment, demonstrating that subgenome expression bias is not static but dynamically adjusted in response to environmental cues. Although most triads retained their original states (MB or MU) under both NaCl and hormone treatments, approximately one-third of triads showed continuous changes in bias type (Figure 6E).

We then analyzed these dynamics in relation to duplication models (Figure 6H). Most triads maintained their original expression bias type before and after treatment, indicating a degree of stability in subgenome partitioning. However, triads derived from different evolutionary models showed distinct patterns of plasticity. For example, triads from model II (especially in group S) and model III (especially in group F) were more prone to switching triad type, suggesting that evolutionary origin influences the expression flexibility.

In addition, some triads underwent changes in the identity of the dominant subgenome under specific conditions. For instance, in model I, the triad in clade7 (*TraesCS3A03G0894700*, *TraesCS3B03G1020000*, and *TraesCS3D03G0821900*) shifted from B-dominant before NaCl treatment to D-dominant after treatment, whereas it remained B-dominant under hormone treatments, indicating condition-dependent subgenome dominance. Conversely, certain triads showed a rebalancing effect. For example, in model II, clade43 triads that were initially unbalanced became balanced after NaCl treatment, suggesting that environmental stimuli may promote coordinated expression among subgenomes.

These results show that subgenome expression bias in wheat *bZIP* triads displays both stability and plasticity. Although duplication history contributes to the propensity for biased expression, dynamic reconfiguration under stress and hormone treatments is more likely driven by post-duplication regulatory divergence than by duplication origin alone.

### 2.7. Variation in TE Composition Among Representative bZIP Clades and Subgenomes

**Figure 7:**
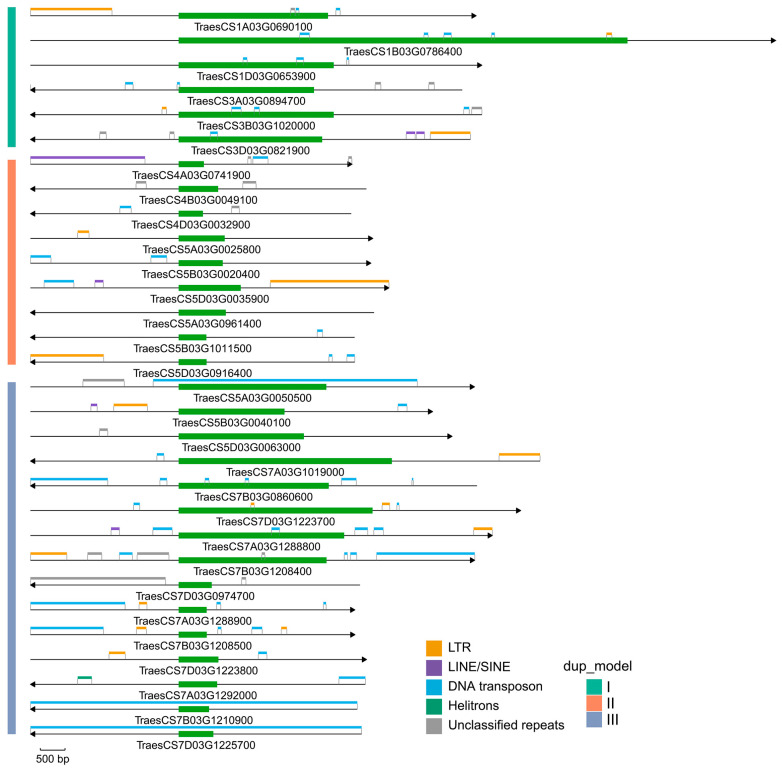
Transposable element (TE) landscapes in the ±3 kb flanking regions of *bZIP* genes from different duplication models in wheat. Schematic representation of TE distributions in the genomic regions spanning ±3 kb of representative *bZIP* genes from three duplication models: model I (clade 7), model II (clade 43), and model III (clade 27). Each horizontal line represents a gene locus, with arrows indicating gene orientation. Green boxes denote *bZIP* gene bodies. Colored blocks indicate different classes of TE.

To explore potential genomic factors underlying transcriptional divergence and subgenome expression bias, we selected three representative clades for detailed comparative analysis, including clade 7 (model I), clade 43 (model II), and clade 27 (model III) (Figure 7). Across these clades, TE distributions varied substantially in both composition and abundance. Multiple TE classes, including LTR retrotransposons, LINE/SINE elements, DNA transposons, and Helitrons, were detected in gene bodies and ±3 kb flanking regions. Differences in TE composition were observed among the three representative duplication models. Genes in model I (clade 7) generally exhibited relatively similar TE patterns among homoeologous copies, whereas genes in model II (clade 43) and model III (clade 27) showed more heterogeneous TE distributions. In addition, homoeologous copies from the A, B, and D subgenomes frequently displayed distinct TE insertions and TE densities in their flanking regions.

Although this analysis was limited to representative clades rather than genome-wide comparisons, the observed variation in TE composition and distribution suggests that duplicated *bZIP* genes from different evolutionary backgrounds may reside in distinct genomic environments.

## 3. Discussion

### 3.1. Duplication Landscapes Reveal Constrained Yet Biased Retention of bZIP Genes

Our analyses show that the expansion of the bZIP family in Pooideae cannot be understood simply from genome-wide duplication composition. Although SSD, particularly DD, dominate genome-wide gene repertoires, WGD/segmental duplication consistently represents the major source of *bZIP* genes across species. This contrast indicates that duplication modes do not contribute equally to the *bZIP* family evolution and suggests strong retention bias rather than passive inheritance from genomic background alone.

Correlation analyses further indicate that genome-wide duplication patterns impose a baseline constraint on gene-family-level composition. The positive relationship between genome-wide and *bZIP* family-level WGD proportions suggests that the availability of WGD/segmental-derived genes is partly determined by the genomic background. However, this constraint alone cannot fully explain the observed duplication bias. Hypergeometric enrichment analyses show that WGD/segmental-derived *bZIP* genes are consistently overrepresented, whereas DD-derived *bZIP* genes are generally depleted, indicating that retention after duplication is non-random.

These contrasting patterns observed for WGD/segmental and DD suggest that different duplication modes may contribute unequally to the evolutionary composition of the *bZIP* family. WGD/segmental-derived duplicates are consistently enriched within the *bZIP* family relative to genome-wide patterns, whereas dispersed duplicates are comparatively less represented despite their abundance at the genome level. One possible explanation for this pattern is the dosage balance hypothesis, which proposes that genes encoding transcriptional regulators are more likely to be retained following whole-genome duplication in order to maintain stoichiometric relationships within regulatory networks [44,45,46]. However, the current study does not directly evaluate dosage sensitivity or regulatory network architecture, and further analyses will be required to test this possibility more rigorously.

At the subfamily level, duplication bias is also structured rather than random. The predominance of WGD/segmental duplication across most *bZIP* groups (groups A, D, I and S), together with its relative consistency across species, indicates that preferential retention is conserved within the *bZIP* family. The elevated representation of WGD/segmental-derived genes in certain groups further suggests that retention strength varies among subfamilies, likely reflecting in functional constraints.

Importantly, Ks analyses suggest that many retained WGD/segmental-derived *bZIP* genes are associated with the ancient grass-specific ρ-WGD event. The overlap between major Ks peaks and the expected range of the ρ-WGD signal across multiple species is consistent with the possibility that ancient whole-genome duplication contributed substantially to the establishment of the ancestral *bZIP* repertoire prior to the divergence of major grass lineages. Subsequent lineage-specific retention, loss, and expansion likely further shaped the extant *bZIP* composition observed in Pooideae.

These findings support a model in which genome-wide duplication history influences the pool of available duplicates, whereas differential retention contributes to the observed enrichment of WGD/segmental-derived *bZIP* genes across species. Although genome assembly and annotation quality may influence absolute gene numbers in some species, the overall enrichment patterns and Ks distributions were broadly consistent across independently assembled grass genomes. Together, these processes may have shaped the duplication architecture and evolutionary diversification of the *bZIP* gene family in Pooideae.

### 3.2. Divergent Evolutionary Fates of bZIP Genes After Duplication

The identification of four distinct evolutionary models highlights the heterogeneous nature of the *bZIP* family evolution. Rather than following a single path of expansion, different clades experienced different combinations of ancient duplication, lineage-specific retention, additional expansion, and loss. Clades belonging to model I show that ancient duplication events, most likely associated with WGD in the common ancestor of Poaceae, established stable gene lineages that were broadly retained across descendant species. The additional expansion observed in *Triticeae* suggests that these ancestral lineages remained evolutionarily active and continued to diversify after the initial duplication event. In contrast, model II illustrates that ancestral duplication was followed by uneven retention and secondary lineage-specific expansion. The resulting gene repertoires therefore reflect both preservation and pruning of ancestral duplicates. Model III emphasizes the importance of lineage-specific duplication in generating gene diversity. Because these duplications are largely restricted to *Triticeae*, they likely represent lineage-dependent processes that contributed to local adaptation or specialization after divergence from other grasses. Such lineage-restricted expansion may provide opportunities for functional diversification in specific evolutionary contexts. In contrast, clades classified as model IV were retained predominantly as single-copy genes across the examined species, with no detectable duplication events identified in either phylogenetic or syntenic analyses, indicating strong evolutionary constraints on duplication retention. These genes may be subject to dosage sensitivity or functional constraints that limit their duplication, representing a conserved core component of the *bZIP* gene family.

These results support a hierarchical evolutionary framework in which ancient WGD established the basic family backbone, and subsequent lineage-specific duplications generated additional diversity in particular taxa. This multi-layered process underlies the diversity and composition of the *bZIP* family across Pooideae.

### 3.3. Evolutionary Origin Shapes Transcriptional Diversification of bZIP Genes

Because *bZIP* genes are central regulators of development and stress adaptation, their diversification is not reflected only in copy number but also in transcriptional deployment. Our transcriptomic analyses reveal that expression divergence within the family is extensive but also highly structured. These differences are not randomly distributed but emerge progressively across evolutionary levels, from duplication model to phylogenetic group, clade, and individual genes, indicating that duplication history establishes a broad framework, whereas subsequent divergence refines regulatory outcomes.

Importantly, duplication history alone does not determine expression pattern. Although genes within the same duplication model share a common origin, they often diverge substantially at the group and clade levels. This suggests that regulatory evolution after duplication plays a critical role in shaping functional differentiation. In this context, phylogenetic groups and clades appear to be more functionally informative than duplication models alone, as they capture finer-scale divergence in transcriptional behavior.

The association between developmental expression breadth and stress responsiveness provides further insight into the functional organization of the *bZIP* family. Genes that are broadly expressed across tissues tend to respond to a wider range of environmental stimuli, suggesting that they may act as core regulators integrating developmental and stress-related signals. In contrast, genes with restricted developmental expression profiles more often exhibit condition-specific responses, consistent with increasing specialization after duplication.

Notably, broadly expressed and broadly responsive genes are preferentially enriched in particular evolutionary lineages. Group I genes, which are exclusively derived from model I, represent a highly conserved lineage that retains both broad expression and broad responsiveness, suggesting a central regulatory role. Group A genes show a similar but slightly more diversified pattern, whereas group S genes, primarily derived from model II, appear to reflect a more specialized trajectory shaped by duplication followed by differential retention and divergence.

These observations support a model in which different duplication histories contribute to distinct functional strategies. Genes derived from ancient duplication events (model I), especially those retained across lineages, tend to form a conserved regulatory core, whereas genes shaped by lineage-specific retention, loss, or more recent duplication (model II) are more likely to contribute to specialized transcriptional responses.

### 3.4. Stability and Plasticity of Subgenome Expression Partitioning in Polyploid Triticeae

In polyploid *Triticeae*, transcriptional diversification of *bZIP* genes is further shaped by subgenome partitioning. By integrating developmental and stress/hormone-responsive transcriptome data, we demonstrate that subgenome expression bias of *bZIP* homoeologous triads in wheat exhibits a dual property of stability and plasticity. Although this study focuses on wheat as a representative species, the evolutionary processes underlying *bZIP* expansion including ancient whole-genome duplication, lineage-specific expansion, and recent polyploidization are shared across *Triticeae* species. Therefore, the patterns observed here are likely reflective of a principle governing homoeolog expression in polyploid *Triticeae* rather than being unique to wheat.

During development, a large proportion of triads maintain consistent expression bias patterns, indicating that subgenome expression partitioning established after polyploidization is relatively stable under normal growth conditions. This stability is partially retained under stress and hormone treatments, as most triads preserve their original expression types, suggesting that subgenome bias forms a robust transcriptional framework. At the same time, this framework is not rigid. Under stress and hormone treatments, approximately one-third of triads change bias type, and in some cases the dominant subgenome switches depending on the condition. This indicates that subgenome expression bias is not a fixed property, but a dynamic state that can be modulated in response to environmental signals. Notably, hormone treatments induce broader reconfiguration than NaCl treatment, suggesting a stronger capacity to globally reshape homoeolog contribution.

The observed changes are strongly condition-dependent. The same triad can behave differently under different treatments, implying that distinct signaling pathways influence subgenome partitioning through different regulatory mechanisms. This condition-dependent behavior suggests that subgenome bias represents an active layer of regulatory flexibility rather than merely a passive consequence of genome merger.

Duplication history contributes to, but does not fully determine, these expression patterns. Although certain duplication models (e.g., model I) show a higher tendency toward biased expression, and others (e.g., model II and III) display distinct degrees of plasticity, triads within the same model, group, and clade can still behave differently. This highlights the importance of post-duplication regulatory divergence, including changes in cis-regulatory elements, transcriptional networks, and epigenetic states, in shaping homoeolog expression dynamics [8,36,47,48].

These findings support a unified model in which polyploidization establishes a baseline framework of gene retention and expression partitioning, while subsequent divergence enables dynamic and condition-dependent redistribution of transcriptional contributions among subgenomes.

### 3.5. Exploratory Analysis of TE Variation in Representative bZIP Clades

The exploratory TE analysis presented here suggests that duplicated *bZIP* genes from different evolutionary backgrounds may be associated with distinct local genomic contexts. Across the three representative clades examined, differences in TE composition and abundance were observed both among duplication models and among homoeologous copies from different subgenomes. In particular, model II and III clades exhibited more heterogeneous TE distributions than the representative model I clade, paralleling the greater expression divergence observed in these groups (Figure 6H and Table S8).

However, because this analysis was based on a limited number of representative clades rather than a genome-wide quantitative comparison, the current results do not establish a direct mechanistic relationship between TE composition and expression divergence or subgenome expression bias. Instead, they provide preliminary observations suggesting that variation in local genomic environments may be associated with transcriptional differentiation among duplicated *bZIP* genes.

Previous studies have shown that TE insertions can influence nearby gene expression through effects on chromatin organization or cis-regulatory landscapes [8,49,50,51]. In this context, the asymmetric TE distributions observed among homoeologous copies may represent one potential factor associated with the unequal expression contributions detected among subgenomes. Nevertheless, further genome-wide analyses and functional validation will be required to determine whether specific TE classes or TE abundances are quantitatively linked to the magnitude or direction of expression divergence.

Taken together, these representative observations suggest that local genomic context may vary substantially among duplicated *bZIP* genes and among subgenomes in polyploid Triticeae. Although exploratory in nature, these patterns provide a useful framework for future investigation of the relationship between TE landscapes and transcriptional diversification following gene duplication.

## 4. Materials and Methods

### 4.1. Retrieval of Sequences

Genome assemblies and published annotation versions used in this study are summarized in Table S1. All *bZIP* genes from the sampled species were retrieved following a previously described pipeline [52,53]. bZIP protein sequences from *A. thaliana* [14] were used to search for homologs by BLASTP or TBLASTN program in ncbi-blast v2.14. 0 with an E value of <1 × 10^−5^. For wheat, we selected the T2T assembly and annotation for the gene identification and all downstream analyses.

### 4.2. Multiple Sequence Alignments

Sequences were aligned using MAFFT v7.313 (L-INS-i option) with 1000 iterations [54]. The resulting alignments were subsequently inspected, manually adjusted, and refined using trimAl v1.2 [55]. Regions containing excessive gaps or ambiguous sites were excluded, applying a minimum coverage threshold of 60%.

### 4.3. Phylogenetic Analyses

The optimal substitution models were determined with ProtTest v3.4.2 [56] based on the Bayesian Information Criterion (BIC). Maximum likelihood phylogenetic trees were inferred using IQ-TREE v1.6.8 [57]. Final consensus trees were visualized and annotated using ggtree [58]. Detailed information on model selection and iteration parameters is provided in the corresponding figure.

### 4.4. Gene Clade Inference

*bZIP* clade delineation was performed by integrating orthology inference with gene clustering patterns observed in the maximum-likelihood trees. Orthology inference was performed using OrthoFinder v2.3 [59] to calibrate the gene grouping results. Monophyletic clades were identified based on consistent clustering of genes within both orthogroup assignment and phylogenetic topology. Evolutionary model assignment was then inferred according to the phylogenetic positions and relative timing of duplication events observed within each clade.

### 4.5. Gene Duplication and Synteny Analysis

Gene duplication patterns and syntenic relationships of *bZIP* family members were investigated using MCScanX v1.0.0 [60]. Protein sequences from 16 Poaceae genomes were subjected to all-vs.-all similarity searches using BLASTP separately with an E-value cutoff of 1 × 10^−5^. To ensure reliable homolog detection, only the top 5 ranking hits were retained for downstream processing. The BLASTP results, together with gene position information derived from GFF3 files (converted into BED format), were used as input for MCScanX to identify collinear blocks within genomes. Syntenic gene pairs located within these collinear regions were considered as candidates for whole-genome or segmental duplication events. Additionally, duplication types, including tandem, proximal, dispersed, and segmental duplications, were classified using the duplicate_gene_classifier module implemented in MCScanX. Duplicate genes were classified according to the hierarchical criteria implemented in MCScanX. When a gene pair satisfied multiple duplication categories, classification priority followed the default MCScanX order: WGD/segmental > tandem > proximal > dispersed. Therefore, genes located within syntenic collinear blocks were preferentially assigned as WGD/segmental duplicates rather than dispersed duplicates.

### 4.6. Ks Estimates

Syntenic gene pairs identified by MCScanX were used for Ks analysis. Protein sequences of each duplicated gene pair were first aligned using MAFFT v7.313 [54] with default parameters. The corresponding coding sequences were then aligned based on the protein alignment using PAL2NA v14 [61]. Ks values were calculated using the codeml program implemented in PAML v4.9j [62] under the F3×4 codon frequency model. Only gene pairs with Ks values between 0 and 3 were retained for downstream analyses to avoid saturation effects.

### 4.7. Statistical Analysis

Spearman correlation coefficients were calculated using R (v4.6.0). Statistical significance was evaluated using two-tailed tests. Hypergeometric tests were performed using the phyper function in R. Contingency-table-based Fisher’s exact tests were used to evaluate associations between duplication models and subgenome expression-bias categories, as well as enrichment patterns across phylogenetic groups. *p*-values were adjusted for multiple testing using the Benjamini–Hochberg method, and adjusted *p*-values < 0.05 were considered statistically significant.

### 4.8. Transcriptome Analyses

Transcriptome data for 5 tissues (leaf, root, shoot, spike and grain) of wheat genomes were retrieved from the published study [63]. Raw transcriptome data for stress and hormone treatment were downloaded with the accession number listed in Table S9. All RNA-seq datasets were processed using a unified analysis pipeline, including consistent procedures for read preprocessing, genome alignment, and gene expression quantification. For samples with biological replicates, mean TPM values and standard deviations (SDs) were calculated for each gene within each condition or tissue. To improve cross-dataset comparability among datasets generated from different studies, batch effects were corrected using the ComBat function in R. Mean TPM values were subsequently transformed as log_2_(TPM+1) for downstream analyses and visualization. Expression heatmaps were generated using the pheatmap (v1.0.12) package in R.

To characterize expression patterns across developmental stages, genes were classified into four categories based on their expression profiles. Genes with max(TPM) < 1 across all samples were defined as non-expressed. Genes were considered broadly expressed if TPM ≥ 1 in at least three samples spanning at least three different tissues. Genes with TPM ≥ 1 in at least two samples across two tissues were classified as intermediate. Genes with TPM ≥ 1 in at least two samples but restricted to a single tissue were defined as tissue/development-specific. A TPM threshold of 1 was used to reduce the influence of background-level transcription and because it is commonly applied to define reliably expressed genes in plant transcriptomic analyses. The additional requirements for sample number and tissue coverage were introduced to distinguish broadly expressed genes from genes showing sporadic or tissue-restricted expression. To evaluate the robustness of the classification scheme, alternative TPM thresholds (TPM ≥ 0.5 and TPM ≥ 2) were additionally tested, and the overall expression trends and major conclusions remained qualitatively consistent across thresholds (Figure S3).

For stress and hormone treatments, gene responsiveness was classified into three categories. Broadly responsive genes were defined as those showing induction (TPM ≥ 1 and increased relative to control) in at least three treatment conditions. Stress-specific genes were defined as those responsive in only one condition. Non-responsive genes were defined as those not showing induction under any treatment.

### 4.9. Definition of Homoeolog Expression Bias Categories

Relative expression contributions of the A, B, and D homoeologs within each triad were calculated as the proportion of total triad expression. Triads were classified into seven categories following previously described approaches for wheat homoeolog expression partitioning: balanced, A-dominant, B-dominant, D-dominant, A-suppressed, B-suppressed, and D-suppressed [40].

To reduce potential biases caused by low-expression genes, only triads with a total TPM ≥ 1 across the three homoeologs under a given condition were retained for subgenome expression bias analyses. A triad was considered balanced when the relative expression contributions of the three homoeologs approximated the expected 1:1:1 ratio. Dominant and suppressed categories were assigned based on deviation from the balanced state using Euclidean distance to predefined idealized expression vectors, following established wheat triad classification methods.

To characterize dynamic changes under different conditions, triads were further grouped into three categories following previously described approaches [41]: maintain balanced (MB), maintain unbalanced (MU), and constantly changed (CC). MB triads retained the balanced category across all compared conditions, MU triads consistently retained the same unbalanced category, and CC triads exhibited at least one transition between triad categories across conditions.

### 4.10. Transposable Element (TE) Annotation

Transposable elements were annotated using a combination of homology-based and ab initio approaches. Genome sequences were screened against Repbase (2018) [64] using RepeatMasker v4.2.3 [65], and a species-specific repeat library was constructed with RepeatModeler v2.0.7. LTR retrotransposons were identified using LTR_harvest [66], LTR_finder [67], and LTR_retriever [68]. The resulting libraries were combined for final annotation with RepeatMasker. The curated wheat ClariTeRep TE library [69] was additionally incorporated to improve annotation accuracy.

## Data Availability

The original contributions presented in this study are included in the article. Further inquiries can be directed to the corresponding authors.

## Supplementary Materials

The following supporting information can be downloaded at: https://www.mdpi.com/article/10.3390/plants15111608/s1, Figure S1: Phylogenetic trees of genes from groups A–K and S, respectively. The trees were inferred using the maximum likelihood method in IQ-TREE. 46 clades of Pooideae genes were highlighted using different colors. Scale bars indicate substitutions per site; Figure S2: Expression divergence of barley *bZIP* genes across different tissues. Heatmap shows normalized expression levels (log_2_(TPM+1)) across leaf, root, stem, spike, and grain tissues. Genes are ordered according to phylogenetic clades, with annotations indicating duplication model, group, and clade identity; Figure S3: Robustness analysis of expression classification under different TPM thresholds. Venn diagrams showing the overlap between expression categories and stress-responsive gene sets under three TPM thresholds: (A) TPM ≥ 0.5, (B) TPM ≥ 1, and (C) TPM ≥ 2. The upper panels show overlaps between broadly expressed genes and genes responsive to at least three stress conditions (≥3 stress responsive). The middle panels show overlaps between tissue-specific expressed genes and genes responsive to one stress condition (1 stress responsive). The lower panels show overlaps between non-expressed genes and non-responsive genes. Although the absolute numbers of genes in each category varied with TPM threshold, the overall expression patterns and major trends remained qualitatively consistent across thresholds, supporting the robustness of the classification scheme; Table S1: Summary of *bZIP* gene numbers and genome information across 17 species; Table S2: Detailed information for all identified *bZIP* genes across 17 species; Table S3: Genome-wide and bZIP-specific duplication type statistics; Table S4: Expression profiles of *bZIP* genes across tissues and developmental stages; Table S5: Expression profiles of *bZIP* genes under stress and hormone treatments; Table S6: Subgenome expression bias of *bZIP* triads during development; Table S7: Subgenome expression bias of *bZIP* triads under stress and hormone treatments; Table S8: Expression profiles of representative *bZIP* genes used for TE analysis. Table S9: Datasets of raw transcriptome data.
