# Supplementary material for: Ancient Whole-Genome Duplication and Lineage-Specific Retention Shape the Diversification of bZIP Transcription Factors in Pooideae
Source: Plants (Basel). 2026 May 23;15(11):1608. doi: 10.3390/plants15111608 (PMC13259416; doi:10.3390/plants15111608)

Figure S1. Phylogenetic trees of genes from groups A–K and S, respectively. The trees were inferred using the maximum likelihood method in IQ-TREE. 46 clades of Pooideae genes were highlighted using different colors. Scale bars indicate substitutions per site.

Group A: Clades 1-10

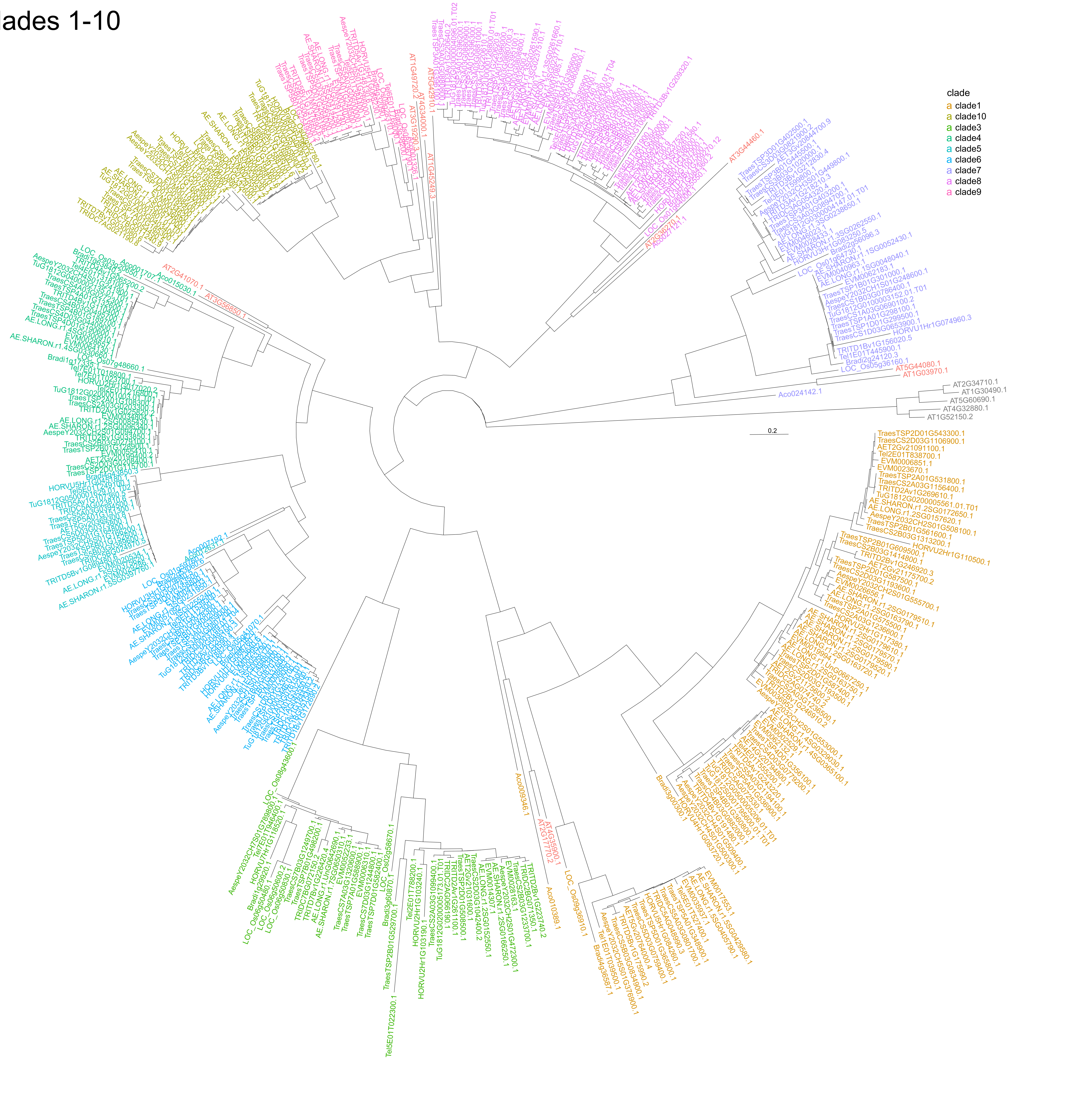

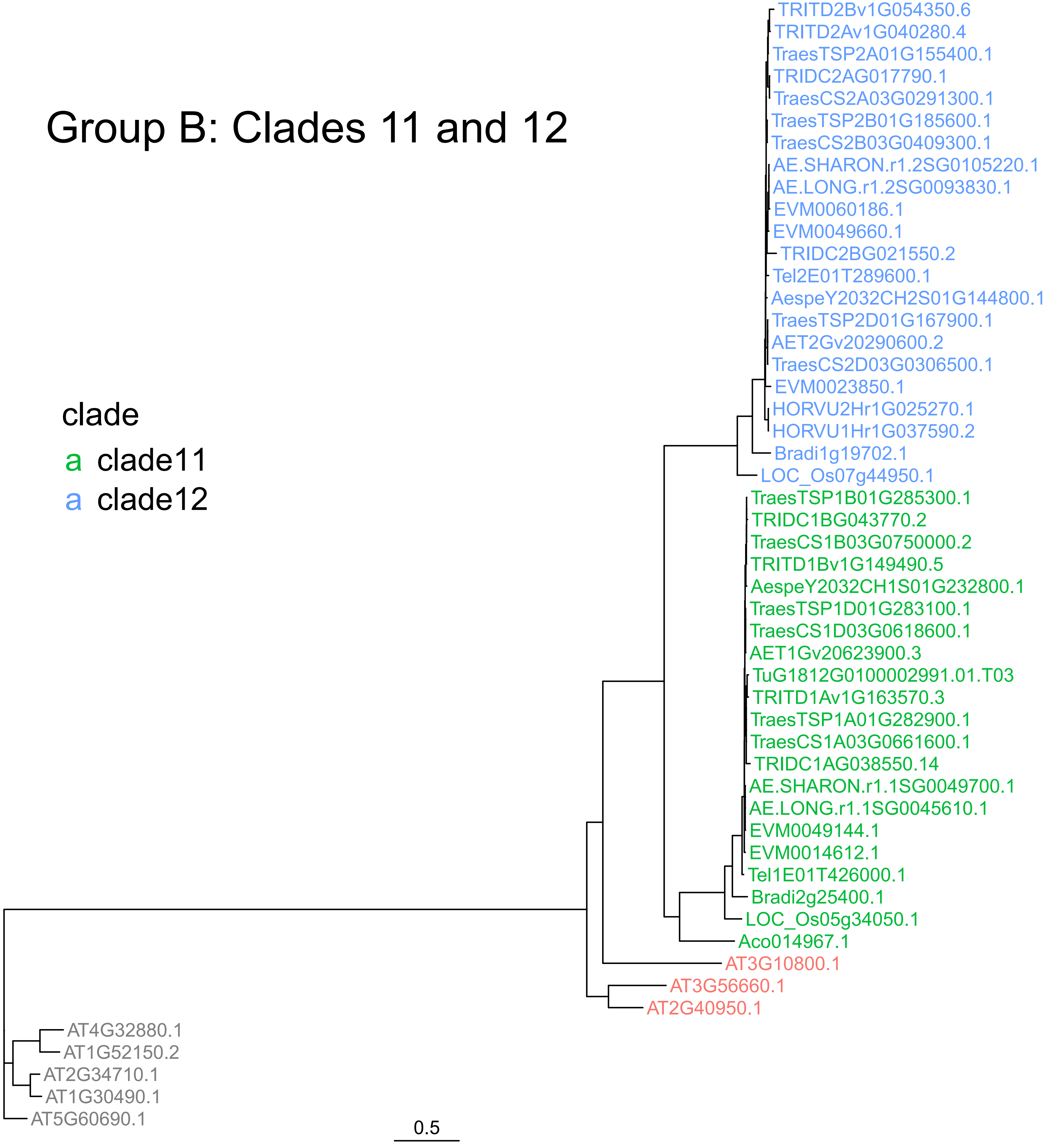
Group B: Clades 11 and 12
clade
a clade11
a clade12
TRITD2Bv1G054350.6
TRITD2Av1G040280.4
TraesTSP2A01G155400.1
TRIDC2AG017790.1
TraesCS2A03G0291300.1
TraesTSP2B01G185600.1
TraesCS2B03G0409300.1
AE.SHARON.r1.2SG0105220.1
AE.LONG.r1.2SG0093830.1
EVM0060186.1
EVM0049660.1
TRIDC2BG021550.2
Tel2E01T289600.1
AespeY2032CH2S01G144800.1
TraesTSP2D01G167900.1
AET2Gv20290600.2
TraesCS2D03G0306500.1
EVM0023850.1
HORVU2Hr1G025270.1
HORVU1Hr1G037590.2
Bradi1g19702.1
LOC_Os07g44950.1
TraesTSP1B01G285300.1
TRIDC1BG043770.2
TraesCS1B03G0750000.2
TRITD1Bv1G149490.5
AespeY2032CH1S01G232800.1
TraesTSP1D01G283100.1
TraesCS1D03G0618600.1
AET1Gv20623900.3
TuG1812G0100002991.01.T03
TRITD1Av1G163570.3
TraesTSP1A01G282900.1
TraesCS1A03G0661600.1
TRIDC1AG038550.14
AE.SHARON.r1.1SG0049700.1
AE.LONG.r1.1SG0045610.1
EVM0049144.1
EVM0014612.1
Tel1E01T426000.1
Bradi2g25400.1
LOC_Os05g34050.1
Aco014967.1
AT3G10800.1
AT3G56660.1
AT2G40950.1
AT4G32880.1
AT1G52150.2
AT2G34710.1
AT1G30490.1
AT5G60690.1
0.5

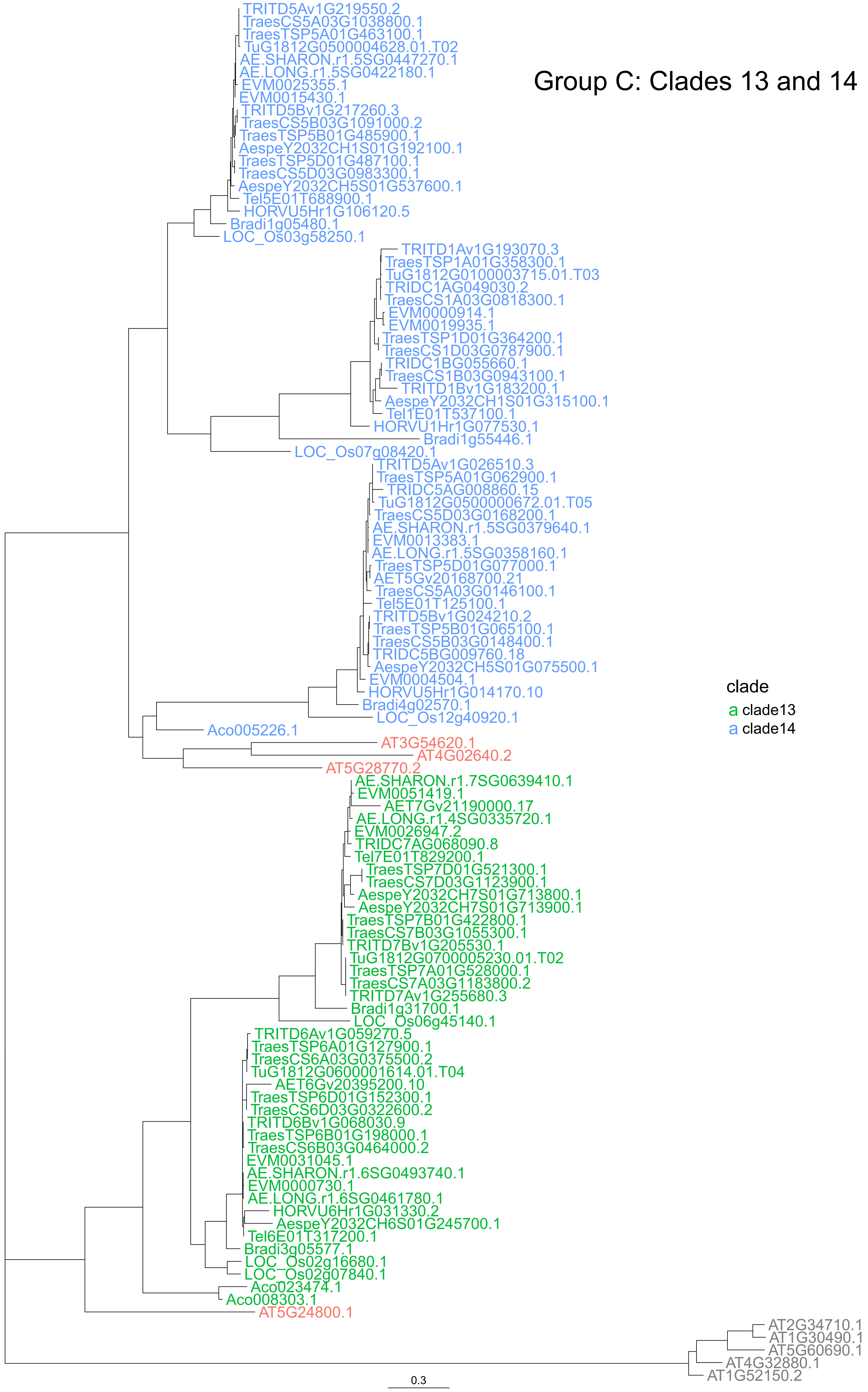
Group C: Clades 13 and 14
TRITD5Av1G219550.2
TraesCS5A03G1038800.1
TraesTSP5A01G463100.1
TuG1812G0500004628.01.T02
AE.SHARON.r1.5SG0447270.1
AE.LONG.r1.5SG0422180.1
EVM0025355.1
EVM0015430.1
TRITD5Bv1G217260.3
TraesCS5B03G1091000.2
TraesTSP5B01G485900.1
AespeY2032CH1S01G192100.1
TraesTSP5D01G487100.1
TraesCS5D03G0983300.1
AespeY2032CH5S01G537600.1
Tel5E01T688900.1
HORVU5Hr1G106120.5
Bradi1g05480.1
LOC_Os03g58250.1
TRITD1Av1G193070.3
TraesTSP1A01G358300.1
TuG1812G0100003715.01.T03
TRIDC1AG049030.2
TraesCS1A03G0818300.1
EVM0000914.1
EVM0019935.1
TraesTSP1D01G364200.1
TraesCS1D03G0787900.1
TRIDC1BG055660.1
TraesCS1B03G0943100.1
TRITD1Bv1G183200.1
AespeY2032CH1S01G315100.1
Tel1E01T537100.1
HORVU1Hr1G077530.1
Bradi1g55446.1
LOC_Os07g08420.1
TRITD5Av1G026510.3
TraesTSP5A01G062900.1
TRIDC5AG008860.15
TuG1812G0500000672.01.T05
TraesCS5D03G0168200.1
AE.SHARON.r1.5SG0379640.1
EVM0013383.1
AE.LONG.r1.5SG0358160.1
TraesTSP5D01G077000.1
AET5Gv20168700.21
TraesCS5A03G0146100.1
Tel5E01T125100.1
TRITD5Bv1G024210.2
TraesTSP5B01G065100.1
TraesCS5B03G0148400.1
TRIDC5BG009760.18
AespeY2032CH5S01G075500.1
EVM0004504.1
HORVU5Hr1G014170.10
Bradi4g02570.1
LOC_Os12g40920.1
Aco005226.1
AT3G54620.1
AT4G02640.2
AT5G28770.2
AE.SHARON.r1.7SG0639410.1
EVM0051419.1
AET7Gv21190000.17
AE.LONG.r1.4SG0335720.1
EVM0026947.2
TRIDC7AG068090.8
Tel7E01T829200.1
TraesTSP7D01G521300.1
TraesCS7D03G1123900.1
AespeY2032CH7S01G713800.1
AespeY2032CH7S01G713900.1
TraesTSP7B01G422800.1
TraesCS7B03G1055300.1
TRITD7Bv1G205530.1
TuG1812G0700005230.01.T02
TraesTSP7A01G528000.1
TraesCS7A03G1183800.2
TRITD7Av1G255680.3
Bradi1g31700.1
LOC_Os06g45140.1
TRITD6Av1G059270.5
TraesTSP6A01G127900.1
TraesCS6A03G0375500.2
TuG1812G0600001614.01.T04
AET6Gv20395200.10
TraesTSP6D01G152300.1
TraesCS6D03G0322600.2
TRITD6Bv1G068030.9
TraesTSP6B01G198000.1
TraesCS6B03G0464000.2
EVM0031045.1
AE.SHARON.r1.6SG0493740.1
EVM0000730.1
AE.LONG.r1.6SG0461780.1
HORVU6Hr1G031330.2
AespeY2032CH6S01G245700.1
Tel6E01T317200.1
Bradi3g05577.1
LOC_Os02g16680.1
LOC_Os02g07840.1
Aco023474.1
Aco008303.1
AT5G24800.1
AT2G34710.1
AT1G30490.1
AT5G60690.1
AT4G32880.1
AT1G52150.2
clade
a clade13
a clade14
0.3

Group D: Clades 15-21

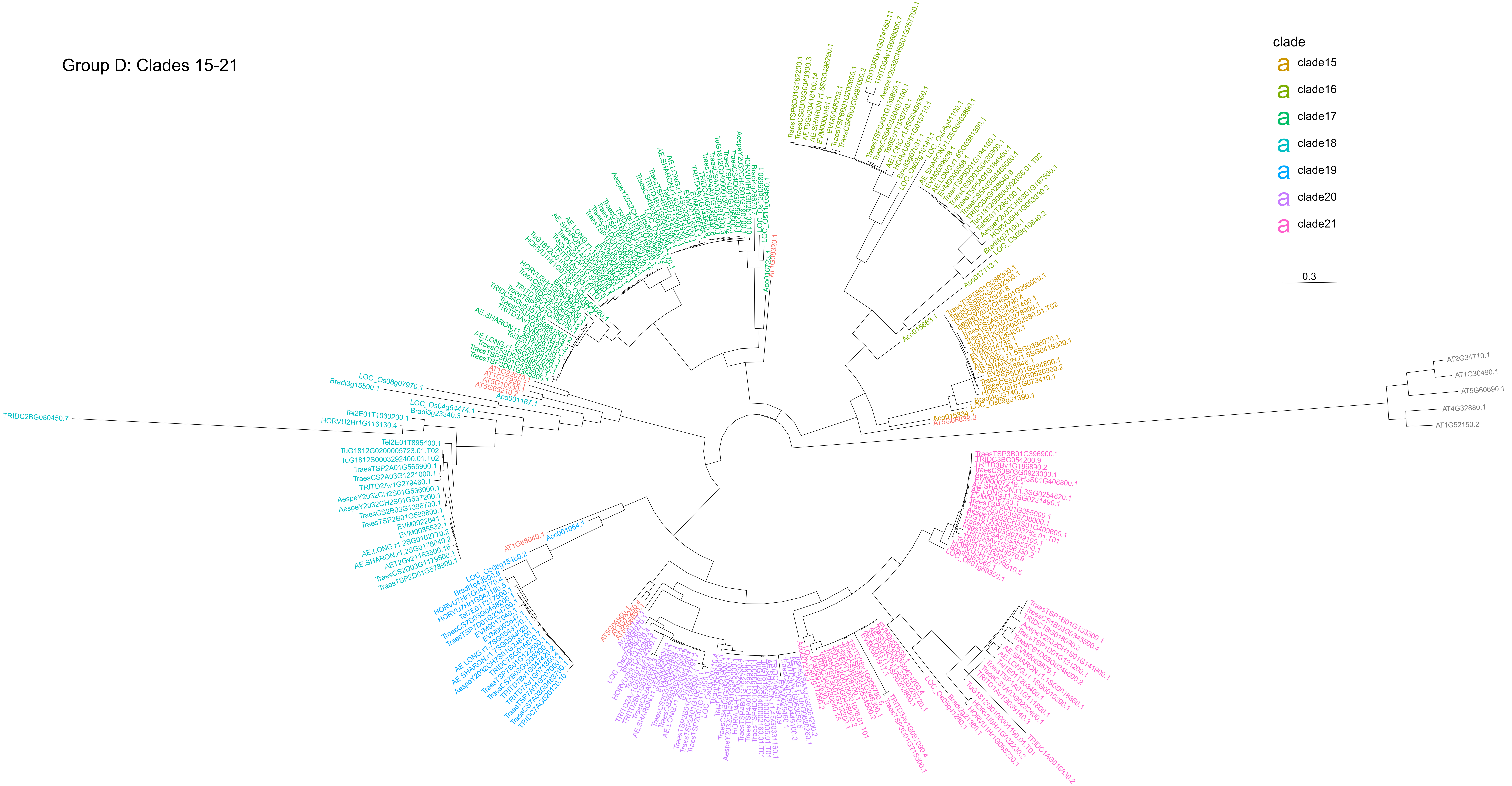

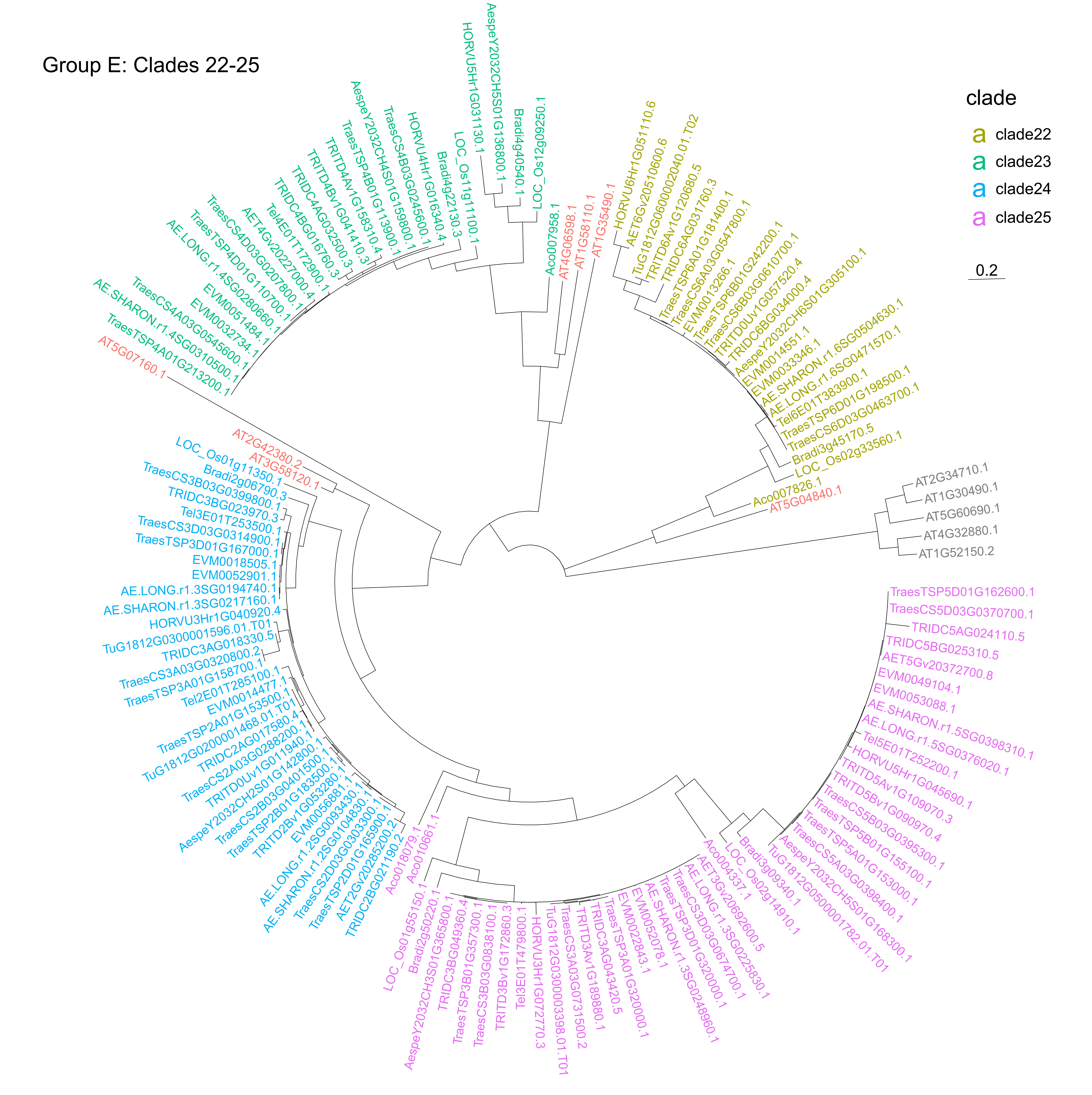

Group E: Clades 22-25
clade
a clade22
a clade23
a clade24
a clade25
0.2
AespeY2032CH5S01G136800.1
HORVU5Hr1G031130.1
Bradi4g40540.1
LOC_Os12g09250.1
LOC_Os11g11100.1
Bradi4g22130.3
HORVU4Hr1G016340.4
TraesCS4B03G0245600.1
AespeY2032CH4S01G159800.1
TraesTSP4B01G113900.1
TRITD4Av1G158310.4
TRITD4Bv1G041410.3
TRIDC4AG032500.3
TRIDC4BG016760.3
Tel4E01T172900.1
AET4Gv20227000.4
TraesCS4D03G0207800.1
TraesTSP4D01G110700.1
AE.LONG.r1.4SG0280660.1
EVM0051484.1
EVM0032734.1
TraesCS4A03G0545600.1
AE.SHARON.r1.4SG0310500.1
TraesTSP4A01G213200.1
AT5G07160.1
Aco007958.1
AT4G06598.1
AT1G58110.1
AT1G35490.1
HORVU6Hr1G051110.6
AET6Gv20510600.6
TuG1812G0600002040.01.T02
TRITD6Av1G120680.5
TRIDC6AG031760.3
TraesTSP6A01G181400.1
TraesCS6A03G0547800.1
EVM0013266.1
TraesTSP6B01G242200.1
TraesCS6B03G0610700.1
TRITD0Uv1G057520.4
TRIDC6BG034000.4
AespeY2032CH6S01G305100.1
EVM0014551.1
EVM0033346.1
AE.SHARON.r1.6SG0504630.1
AE.LONG.r1.6SG0471570.1
Tel6E01T383900.1
TraesTSP6D01G198500.1
TraesCS6D03G0463700.1
Bradi3g45170.5
LOC_Os02g33560.1
Aco007826.1
AT5G04840.1
AT2G34710.1
AT1G30490.1
AT5G60690.1
AT4G32880.1
AT1G52150.2
AT2G42380.2
AT3G58120.1
LOC_Os01g11350.1
Bradi2g06790.3
TraesCS3B03G0399800.1
TRIDC3BG023970.3
Tel3E01T253500.1
TraesCS3D03G0314900.1
TraesTSP3D01G167000.1
EVM0018505.1
EVM0052901.1
AE.LONG.r1.3SG0194740.1
AE.SHARON.r1.3SG0217160.1
HORVU3Hr1G040920.4
TuG1812G0300001596.01.T01
TRIDC3AG018330.5
TraesCS3A03G0320800.2
TraesTSP3A01G158700.1
Tel2E01T285100.1
EVM0014477.1
TraesTSP2A01G153500.1
TuG1812G0200001468.01.T01
TRIDC2AG017580.4
TraesCS2A03G0288200.1
TRITD0Uv1G011940.1
AespeY2032CH2S01G142800.1
TraesCS2B03G0401500.1
TraesTSP2B01G183500.1
TRITD2Bv1G053280.1
EVM0056881.1
AE.LONG.r1.2SG0093430.1
AE.SHARON.r1.2SG0104830.1
TraesCS2D03G0303300.1
TraesTSP2D01G165900.1
AET2Gv20285200.2
TRIDC2BG021190.2
Aco018079.1
Aco010661.1
LOC_Os01g55150.1
Bradi2g50220.1
AespeY2032CH3S01G365800.1
TRIDC3BG049360.4
TraesTSP3B01G357300.1
TraesCS3B03G0838100.1
TRITD3Bv1G172860.3
Tel3E01T479800.1
HORVU3Hr1G072770.3
TuG1812G0300003398.01.T01
TraesCS3A03G0731500.2
TRITD3Av1G189880.1
TRIDC3AG043420.5
TraesTSP3A01G320000.1
EVM0022843.1
EVM0052078.1
AE.SHARON.r1.3SG0248960.1
TraesTSP3D01G320000.1
TraesCS3D03G0674700.1
AE.LONG.r1.3SG0225830.1
AET3Gv20692600.5
Aco004337.1
LOC_Os02g14910.1
Bradi3g09340.1
TuG1812G0500001782.01.T01
AespeY2032CH5S01G168300.1
TraesCS5A03G0398400.1
TraesTSP5A01G153000.1
TraesTSP5B01G155100.1
TraesCS5B03G0395300.1
TRITD5Bv1G090970.4
TRITD5Av1G109070.3
HORVU5Hr1G045690.1
Tel5E01T252200.1
AE.LONG.r1.5SG0376020.1
AE.SHARON.r1.5SG0398310.1
EVM0053088.1
EVM0049104.1
AET5Gv20372700.8
TRIDC5BG025310.5
TRIDC5AG024110.5
TraesCS5D03G0370700.1
TraesTSP5D01G162600.1

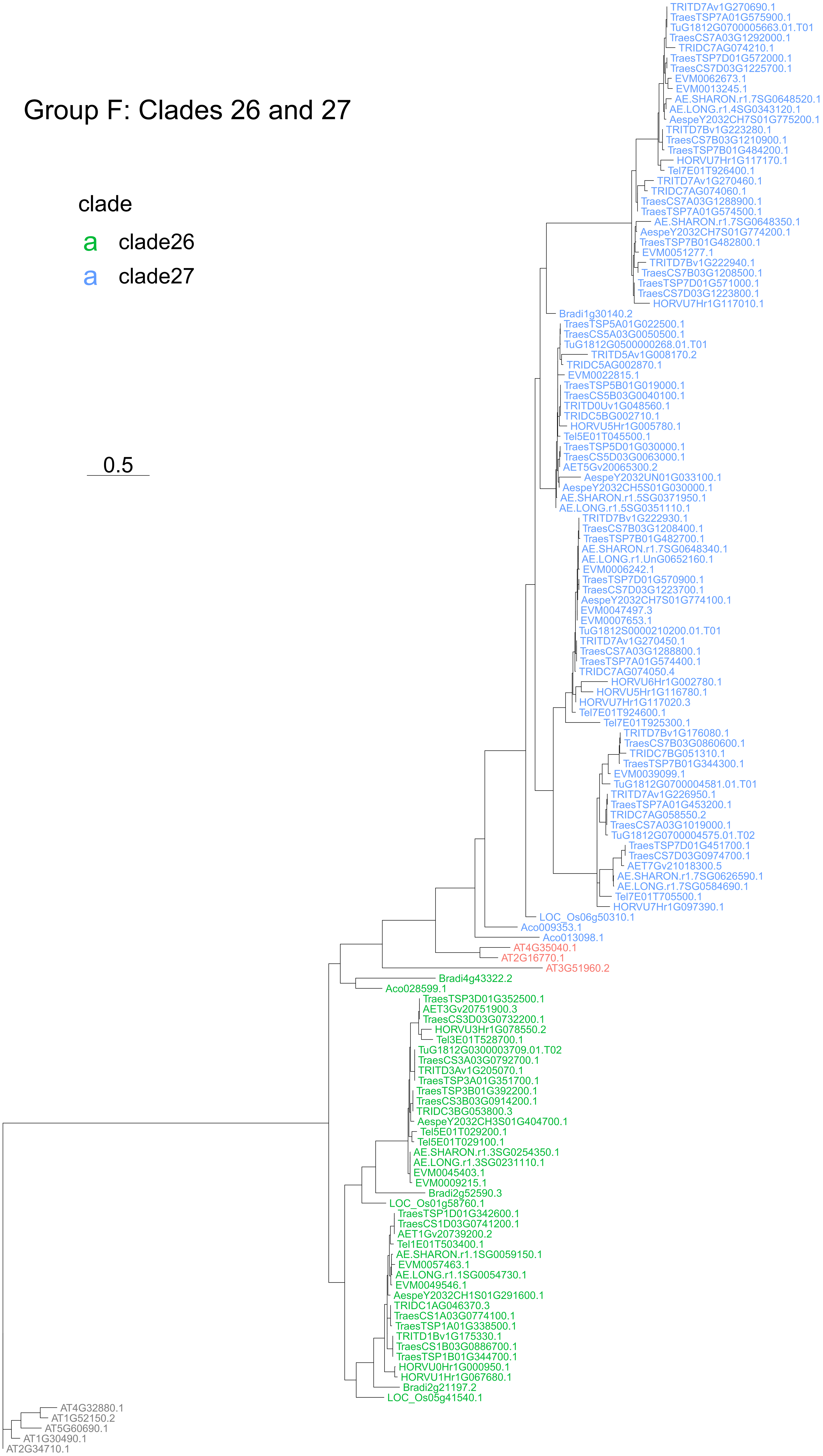

Group F: Clades 26 and 27
clade
a clade26
a clade27
0.5
TRITD7Av1G270690.1
TraesTSP7A01G575900.1
TuG1812G0700005663.01.T01
TraesCS7A03G1292000.1
TRIDC7AG074210.1
TraesTSP7D01G572000.1
TraesCS7D03G1225700.1
EVM0062673.1
EVM0013245.1
AE.SHARON.r1.7SG0648520.1
AE.LONG.r1.4SG0343120.1
AespeY2032CH7S01G775200.1
TRITD7Bv1G223280.1
TraesCS7B03G1210900.1
TraesTSP7B01G484200.1
HORVU7Hr1G117170.1
Tel7E01T926400.1
TRITD7Av1G270460.1
TRIDC7AG074060.1
TraesCS7A03G1288900.1
TraesTSP7A01G574500.1
AE.SHARON.r1.7SG0648350.1
AespeY2032CH7S01G774200.1
TraesTSP7B01G482800.1
EVM0051277.1
TRITD7Bv1G222940.1
TraesCS7B03G1208500.1
TraesTSP7D01G571000.1
TraesCS7D03G1223800.1
HORVU7Hr1G117010.1
Bradi1g30140.2
TraesTSP5A01G022500.1
TraesCS5A03G0050500.1
TuG1812G0500000268.01.T01
TRITD5Av1G008170.2
TRIDC5AG002870.1
EVM0022815.1
TraesTSP5B01G019000.1
TraesCS5B03G0040100.1
TRITD0Uv1G048560.1
TRIDC5BG002710.1
HORVU5Hr1G005780.1
Tel5E01T045500.1
TraesTSP5D01G030000.1
TraesCS5D03G0063000.1
AET5Gv20065300.2
AespeY2032UN01G033100.1
AespeY2032CH5S01G030000.1
AE.SHARON.r1.5SG0371950.1
AE.LONG.r1.5SG0351110.1
TRITD7Bv1G222930.1
TraesCS7B03G1208400.1
TraesTSP7B01G482700.1
AE.SHARON.r1.7SG0648340.1
AE.LONG.r1.UnG0652160.1
EVM0006242.1
TraesTSP7D01G570900.1
TraesCS7D03G1223700.1
AespeY2032CH7S01G774100.1
EVM0047497.3
EVM0007653.1
TuG1812S0000210200.01.T01
TRITD7Av1G270450.1
TraesCS7A03G1288800.1
TraesTSP7A01G574400.1
TRIDC7AG074050.4
HORVU6Hr1G002780.1
HORVU5Hr1G116780.1
HORVU7Hr1G117020.3
Tel7E01T924600.1
Tel7E01T925300.1
TRITD7Bv1G176080.1
TraesCS7B03G0860600.1
TRIDC7BG051310.1
TraesTSP7B01G344300.1
EVM0039099.1
TuG1812G0700004581.01.T01
TRITD7Av1G226950.1
TraesTSP7A01G453200.1
TRIDC7AG058550.2
TraesCS7A03G1019000.1
TuG1812G0700004575.01.T02
TraesTSP7D01G451700.1
TraesCS7D03G0974700.1
AET7Gv21018300.5
AE.SHARON.r1.7SG0626590.1
AE.LONG.r1.7SG0584690.1
Tel7E01T705500.1
HORVU7Hr1G097390.1
LOC_Os06g50310.1
Aco009353.1
Aco013098.1
AT4G35040.1
AT2G16770.1
AT3G51960.2
Bradi4g43322.2
Aco028599.1
TraesTSP3D01G352500.1
AET3Gv20751900.3
TraesCS3D03G0732200.1
HORVU3Hr1G078550.2
Tel3E01T528700.1
TuG1812G0300003709.01.T02
TraesCS3A03G0792700.1
TRITD3Av1G205070.1
TraesTSP3A01G351700.1
TraesTSP3B01G392200.1
TraesCS3B03G0914200.1
TRIDC3BG053800.3
AespeY2032CH3S01G404700.1
Tel5E01T029200.1
Tel5E01T029100.1
AE.SHARON.r1.3SG0254350.1
AE.LONG.r1.3SG0231110.1
EVM0045403.1
EVM0009215.1
Bradi2g52590.3
LOC_Os01g58760.1
TraesTSP1D01G342600.1
TraesCS1D03G0741200.1
AET1Gv20739200.2
Tel1E01T503400.1
AE.SHARON.r1.1SG0059150.1
EVM0057463.1
AE.LONG.r1.1SG0054730.1
EVM0049546.1
AespeY2032CH1S01G291600.1
TRIDC1AG046370.3
TraesCS1A03G0774100.1
TraesTSP1A01G338500.1
TRITD1Bv1G175330.1
TraesCS1B03G0886700.1
TraesTSP1B01G344700.1
HORVU0Hr1G000950.1
HORVU1Hr1G067680.1
Bradi2g21197.2
LOC_Os05g41540.1
AT4G32880.1
AT1G52150.2
AT5G60690.1
AT1G30490.1
AT2G34710.1

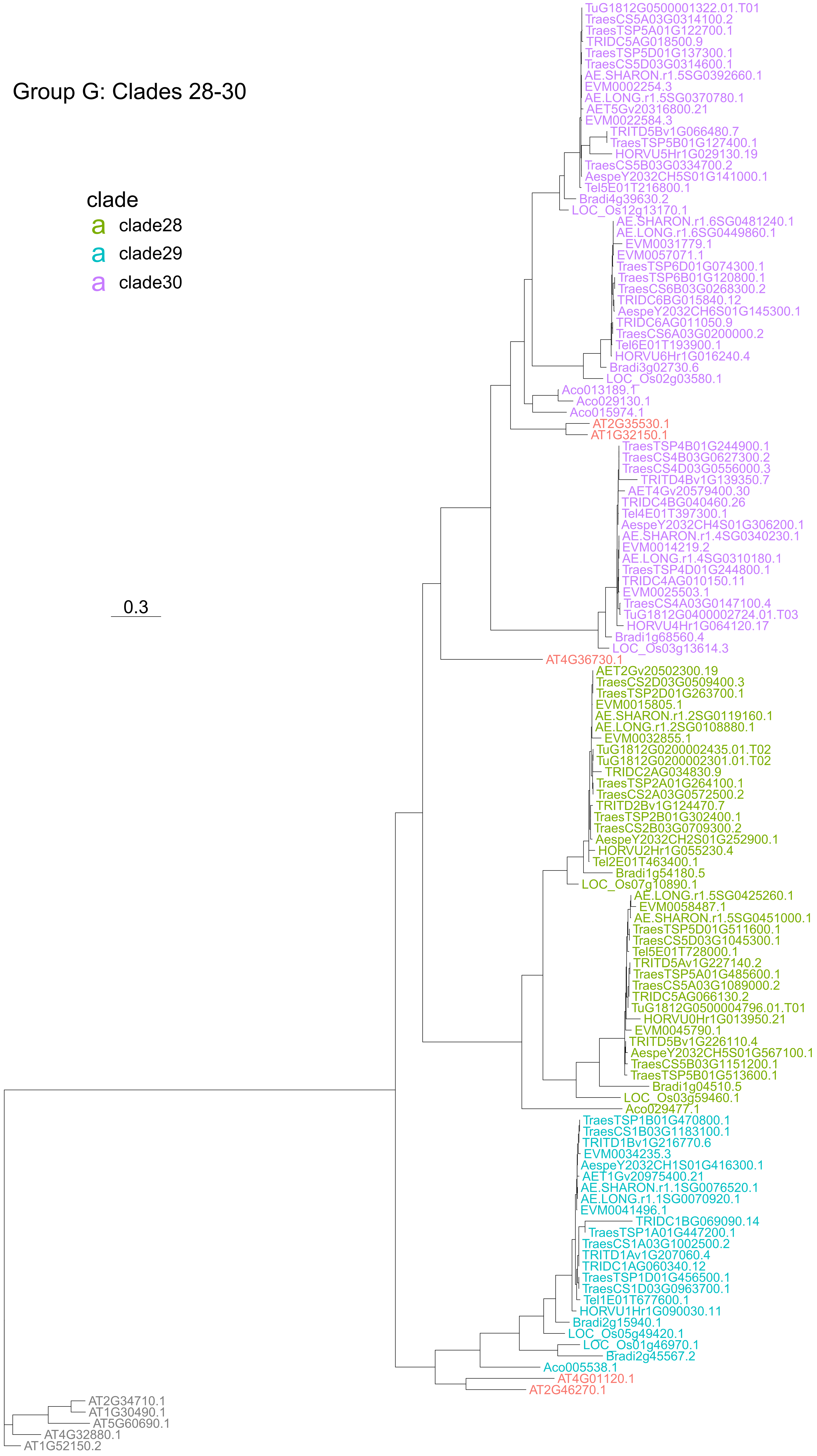
Group G: Clades 28-30
clade
clade28
clade29
clade30
0.3
TuG1812G0500001322.01.T01
TraesCS5A03G0314100.2
TraesTSP5A01G122700.1
TRIDC5AG018500.9
TraesTSP5D01G137300.1
TraesCS5D03G0314600.1
AE.SHARON.r1.5SG0392660.1
EVM0002254.3
AE.LONG.r1.5SG0370780.1
AET5Gv20316800.21
EVM0022584.3
TRITD5Bv1G066480.7
TraesTSP5B01G127400.1
HORVU5Hr1G029130.19
TraesCS5B03G0334700.2
AespeY2032CH5S01G141000.1
Tel5E01T216800.1
Bradi4g39630.2
LOC_Os12g13170.1
AE.SHARON.r1.6SG0481240.1
AE.LONG.r1.6SG0449860.1
EVM0031779.1
EVM0057071.1
TraesTSP6D01G074300.1
TraesTSP6B01G120800.1
TraesCS6B03G0268300.2
TRIDC6BG015840.12
AespeY2032CH6S01G145300.1
TRIDC6AG011050.9
TraesCS6A03G0200000.2
Tel6E01T193900.1
HORVU6Hr1G016240.4
Bradi3g02730.6
LOC_Os02g03580.1
Aco013189.1
Aco029130.1
Aco015974.1
AT2G35530.1
AT1G32150.1
TraesTSP4B01G244900.1
TraesCS4B03G0627300.2
TraesCS4D03G0556000.3
TRITD4Bv1G139350.7
AET4Gv20579400.30
TRIDC4BG040460.26
Tel4E01T397300.1
AespeY2032CH4S01G306200.1
AE.SHARON.r1.4SG0340230.1
EVM0014219.2
AE.LONG.r1.4SG0310180.1
TraesTSP4D01G244800.1
TRIDC4AG010150.11
EVM0025503.1
TraesCS4A03G0147100.4
TuG1812G0400002724.01.T03
HORVU4Hr1G064120.17
Bradi1g68560.4
LOC_Os03g13614.3
AT4G36730.1
AET2Gv20502300.19
TraesCS2D03G0509400.3
TraesTSP2D01G263700.1
EVM0015805.1
AE.SHARON.r1.2SG0119160.1
AE.LONG.r1.2SG0108880.1
EVM0032855.1
TuG1812G0200002435.01.T02
TuG1812G0200002301.01.T02
TRIDC2AG034830.9
TraesTSP2A01G264100.1
TraesCS2A03G0572500.2
TRITD2Bv1G124470.7
TraesTSP2B01G302400.1
TraesCS2B03G0709300.2
AespeY2032CH2S01G252900.1
HORVU2Hr1G055230.4
Tel2E01T463400.1
Bradi1g54180.5
LOC_Os07g10890.1
AE.LONG.r1.5SG0425260.1
EVM0058487.1
AE.SHARON.r1.5SG0451000.1
TraesTSP5D01G511600.1
TraesCS5D03G1045300.1
Tel5E01T728000.1
TRITD5Av1G227140.2
TraesTSP5A01G485600.1
TraesCS5A03G1089000.2
TRIDC5AG066130.2
TuG1812G0500004796.01.T01
HORVU0Hr1G013950.21
EVM0045790.1
TRITD5Bv1G226110.4
AespeY2032CH5S01G567100.1
TraesCS5B03G1151200.1
TraesTSP5B01G513600.1
Bradi1g04510.5
LOC_Os03g59460.1
Aco029477.1
TraesTSP1B01G470800.1
TraesCS1B03G1183100.1
TRITD1Bv1G216770.6
EVM0034235.3
AespeY2032CH1S01G416300.1
AET1Gv20975400.21
AE.SHARON.r1.1SG0076520.1
AE.LONG.r1.1SG0070920.1
EVM0041496.1
TRIDC1BG069090.14
TraesTSP1A01G447200.1
TraesCS1A03G1002500.2
TRITD1Av1G207060.4
TRIDC1AG060340.12
TraesTSP1D01G456500.1
TraesCS1D03G0963700.1
Tel1E01T677600.1
HORVU1Hr1G090030.11
Bradi2g15940.1
LOC_Os05g49420.1
LOC_Os01g46970.1
Bradi2g45567.2
Aco005538.1
AT4G01120.1
AT2G46270.1
AT2G34710.1
AT1G30490.1
AT5G60690.1
AT4G32880.1
AT1G52150.2

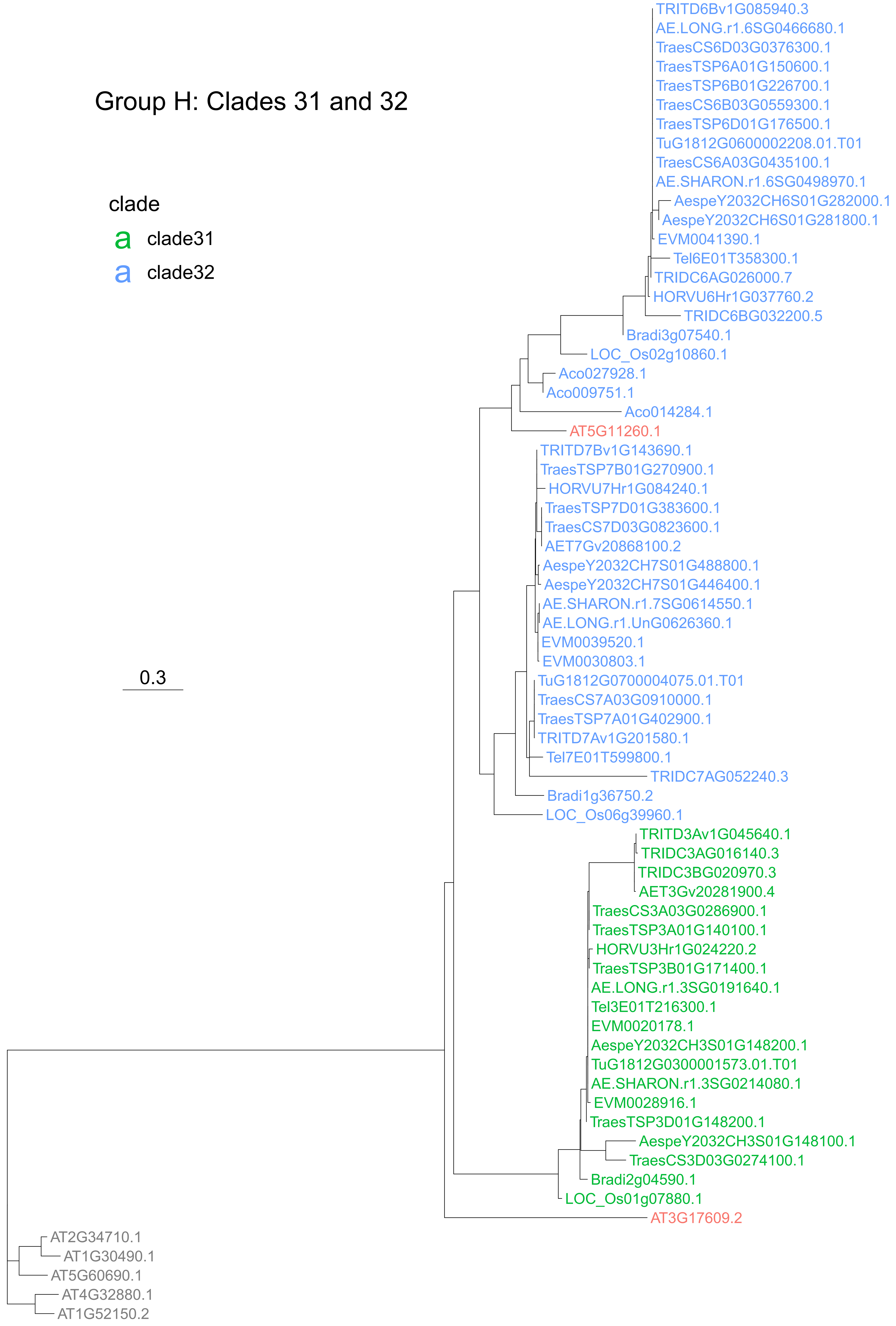
Group H: Clades 31 and 32
clade
clade31
clade32
0.3
TRITD6Bv1G085940.3
AE.LONG.r1.6SG0466680.1
TraesCS6D03G0376300.1
TraesTSP6A01G150600.1
TraesTSP6B01G226700.1
TraesCS6B03G0559300.1
TraesTSP6D01G176500.1
TuG1812G0600002208.01.T01
TraesCS6A03G0435100.1
AE.SHARON.r1.6SG0498970.1
AespeY2032CH6S01G282000.1
AespeY2032CH6S01G281800.1
EVM0041390.1
Tel6E01T358300.1
TRIDC6AG026000.7
HORVU6Hr1G037760.2
TRIDC6BG032200.5
Bradi3g07540.1
LOC_Os02g10860.1
Aco027928.1
Aco009751.1
Aco014284.1
AT5G11260.1
TRITD7Bv1G143690.1
TraesTSP7B01G270900.1
HORVU7Hr1G084240.1
TraesTSP7D01G383600.1
TraesCS7D03G0823600.1
AET7Gv20868100.2
AespeY2032CH7S01G488800.1
AespeY2032CH7S01G446400.1
AE.SHARON.r1.7SG0614550.1
AE.LONG.r1.UnG0626360.1
EVM0039520.1
EVM0030803.1
TuG1812G0700004075.01.T01
TraesCS7A03G0910000.1
TraesTSP7A01G402900.1
TRITD7Av1G201580.1
Tel7E01T599800.1
TRIDC7AG052240.3
Bradi1g36750.2
LOC_Os06g39960.1
TRITD3Av1G045640.1
TRIDC3AG016140.3
TRIDC3BG020970.3
AET3Gv20281900.4
TraesCS3A03G0286900.1
TraesTSP3A01G140100.1
HORVU3Hr1G024220.2
TraesTSP3B01G171400.1
AE.LONG.r1.3SG0191640.1
Tel3E01T216300.1
EVM0020178.1
AespeY2032CH3S01G148200.1
TuG1812G0300001573.01.T01
AE.SHARON.r1.3SG0214080.1
EVM0028916.1
TraesTSP3D01G148200.1
AespeY2032CH3S01G148100.1
TraesCS3D03G0274100.1
Bradi2g04590.1
LOC_Os01g07880.1
AT3G17609.2
AT2G34710.1
AT1G30490.1
AT5G60690.1
AT4G32880.1
AT1G52150.2

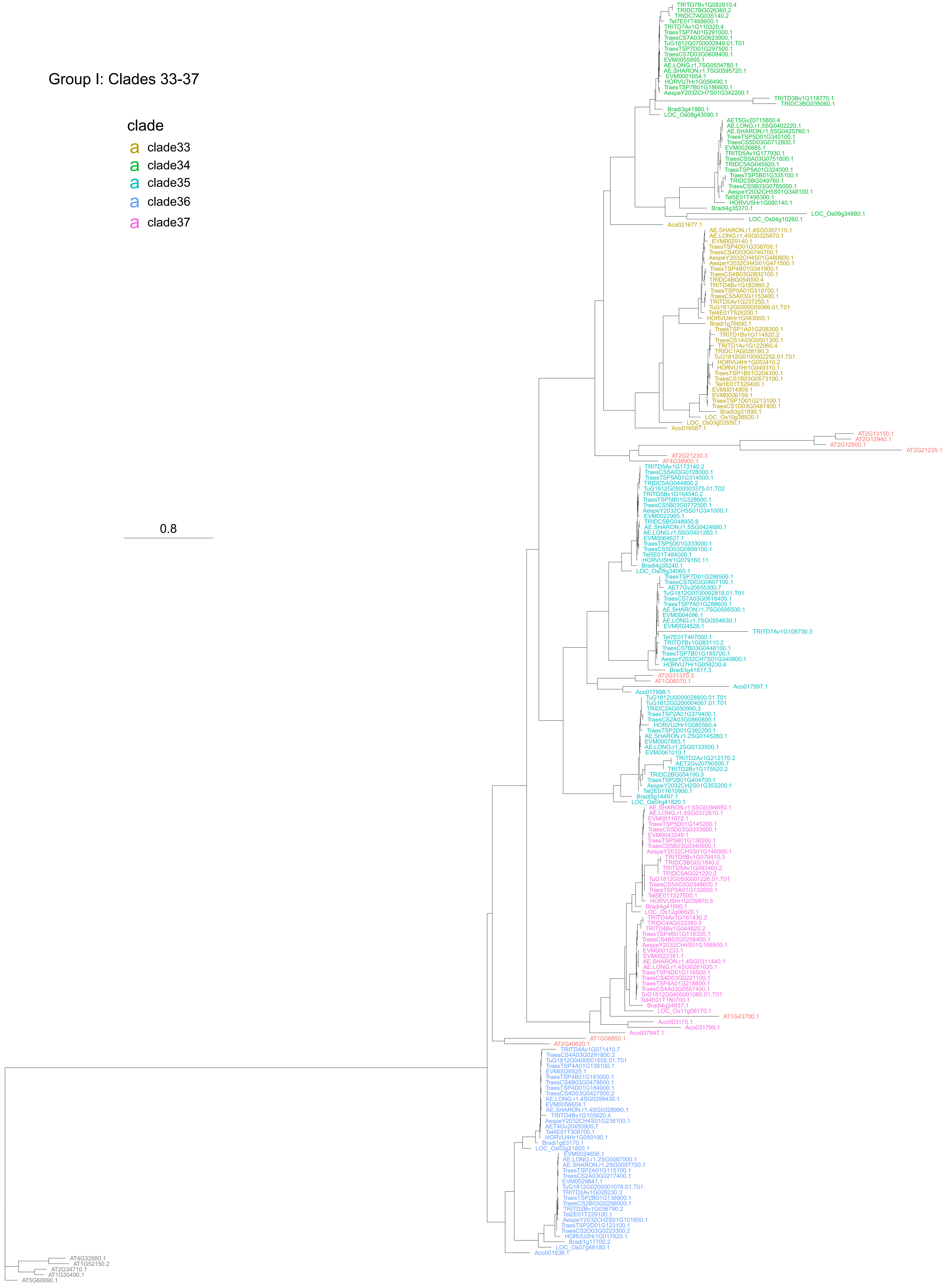

Group I: Clades 33-37
clade
a clade33
a clade34
a clade35
a clade36
a clade37
0.8

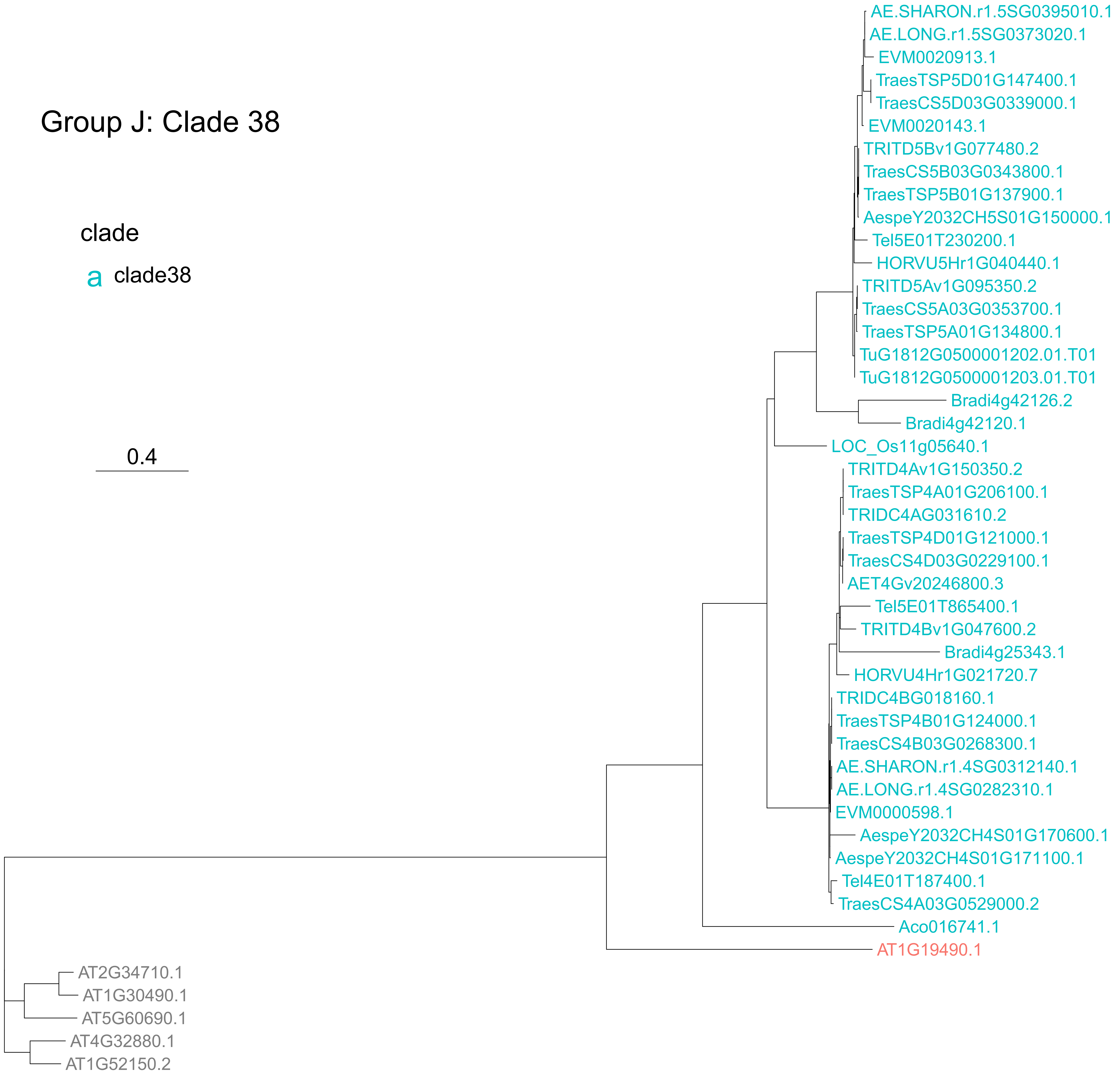

Group J: Clade 38
clade
a clade38
0.4
AE.SHARON.r1.5SG0395010.1
AE.LONG.r1.5SG0373020.1
EVM0020913.1
TraesTSP5D01G147400.1
TraesCS5D03G0339000.1
EVM0020143.1
TRITD5Bv1G077480.2
TraesCS5B03G0343800.1
TraesTSP5B01G137900.1
AespeY2032CH5S01G150000.1
Tel5E01T230200.1
HORVU5Hr1G040440.1
TRITD5Av1G095350.2
TraesCS5A03G0353700.1
TraesTSP5A01G134800.1
TuG1812G0500001202.01.T01
TuG1812G0500001203.01.T01
Bradi4g42126.2
Bradi4g42120.1
LOC_Os11g05640.1
TRITD4Av1G150350.2
TraesTSP4A01G206100.1
TRIDC4AG031610.2
TraesTSP4D01G121000.1
TraesCS4D03G0229100.1
AET4Gv20246800.3
Tel5E01T865400.1
TRITD4Bv1G047600.2
Bradi4g25343.1
HORVU4Hr1G021720.7
TRIDC4BG018160.1
TraesTSP4B01G124000.1
TraesCS4B03G0268300.1
AE.SHARON.r1.4SG0312140.1
AE.LONG.r1.4SG0282310.1
EVM0000598.1
AespeY2032CH4S01G170600.1
AespeY2032CH4S01G171100.1
Tel4E01T187400.1
TraesCS4A03G0529000.2
Aco016741.1
AT1G19490.1
AT2G34710.1
AT1G30490.1
AT5G60690.1
AT4G32880.1
AT1G52150.2

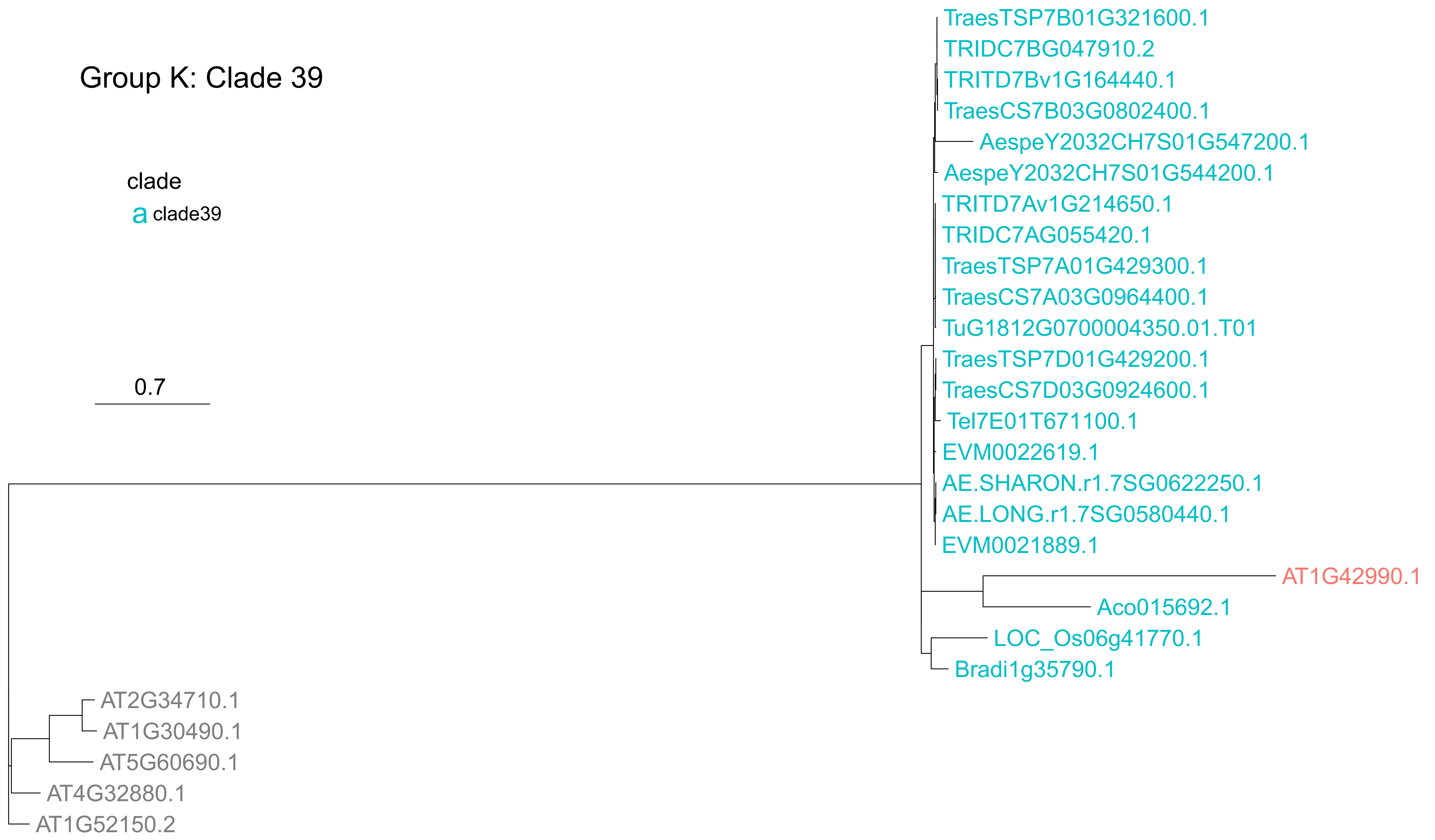
Group K: Clade 39
clade
a clade39
0.7
TraesTSP7B01G321600.1
TRIDC7BG047910.2
TRITD7Bv1G164440.1
TraesCS7B03G0802400.1
AespeY2032CH7S01G547200.1
AespeY2032CH7S01G544200.1
TRITD7Av1G214650.1
TRIDC7AG055420.1
TraesTSP7A01G429300.1
TraesCS7A03G0964400.1
TuG1812G0700004350.01.T01
TraesTSP7D01G429200.1
TraesCS7D03G0924600.1
Tel7E01T671100.1
EVM0022619.1
AE.SHARON.r1.7SG0622250.1
AE.LONG.r1.7SG0580440.1
EVM0021889.1
AT1G42990.1
Aco015692.1
LOC_Os06g41770.1
Bradi1g35790.1
AT2G34710.1
AT1G30490.1
AT5G60690.1
AT4G32880.1
AT1G52150.2

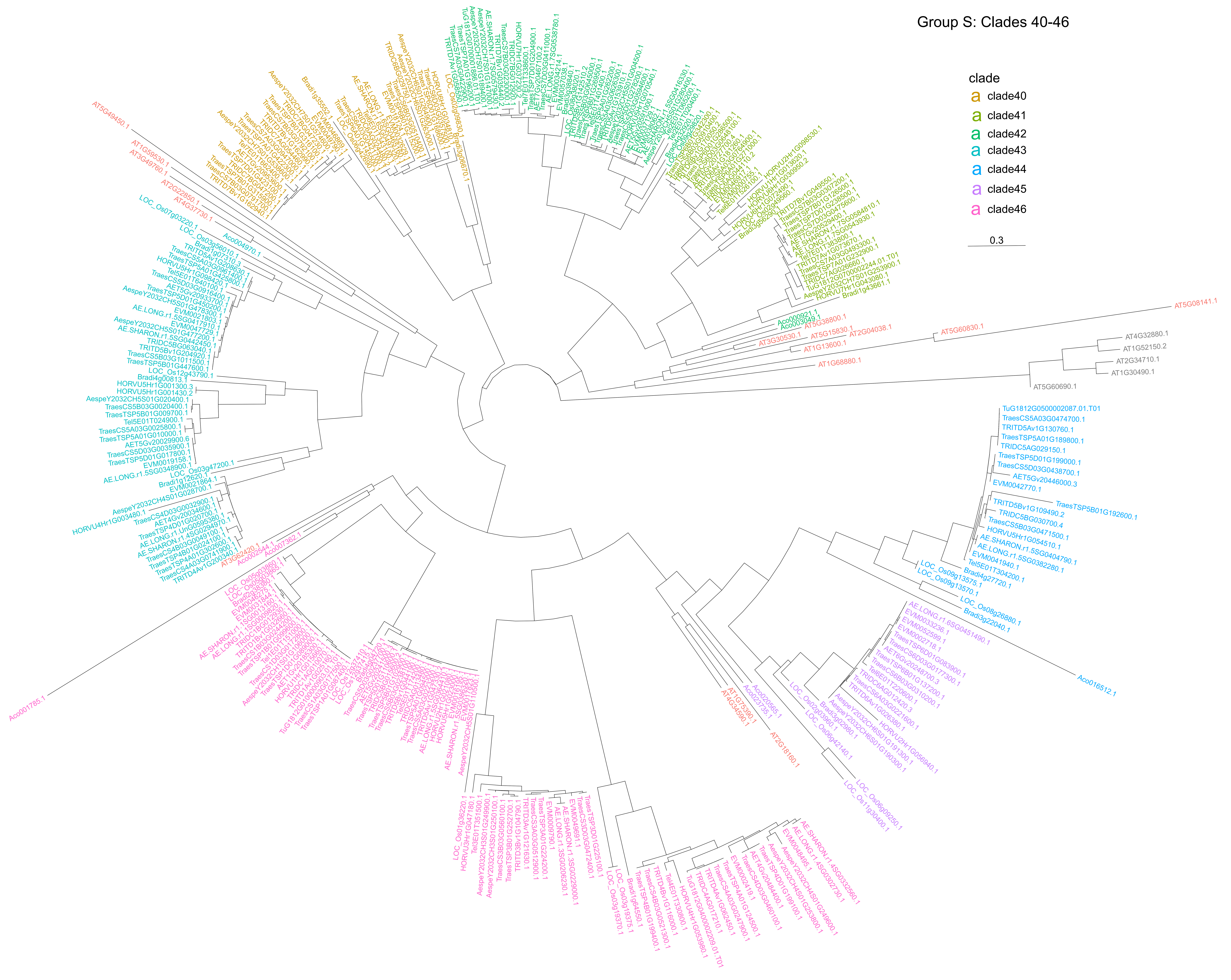
Group S: Clades 40-46
clade
clade40
clade41
clade42
clade43
clade44
clade45
clade46
0.3

Figure S2. Expression divergence of barley *bZIP* genes across different tissues. Heatmap shows normalized expression levels (log_2(TPM+1)) across leaf, root, stem, spike, and grain tissues. Genes are ordered according to phylogenetic clades, with annotations indicating duplication model, group, and clade identity.

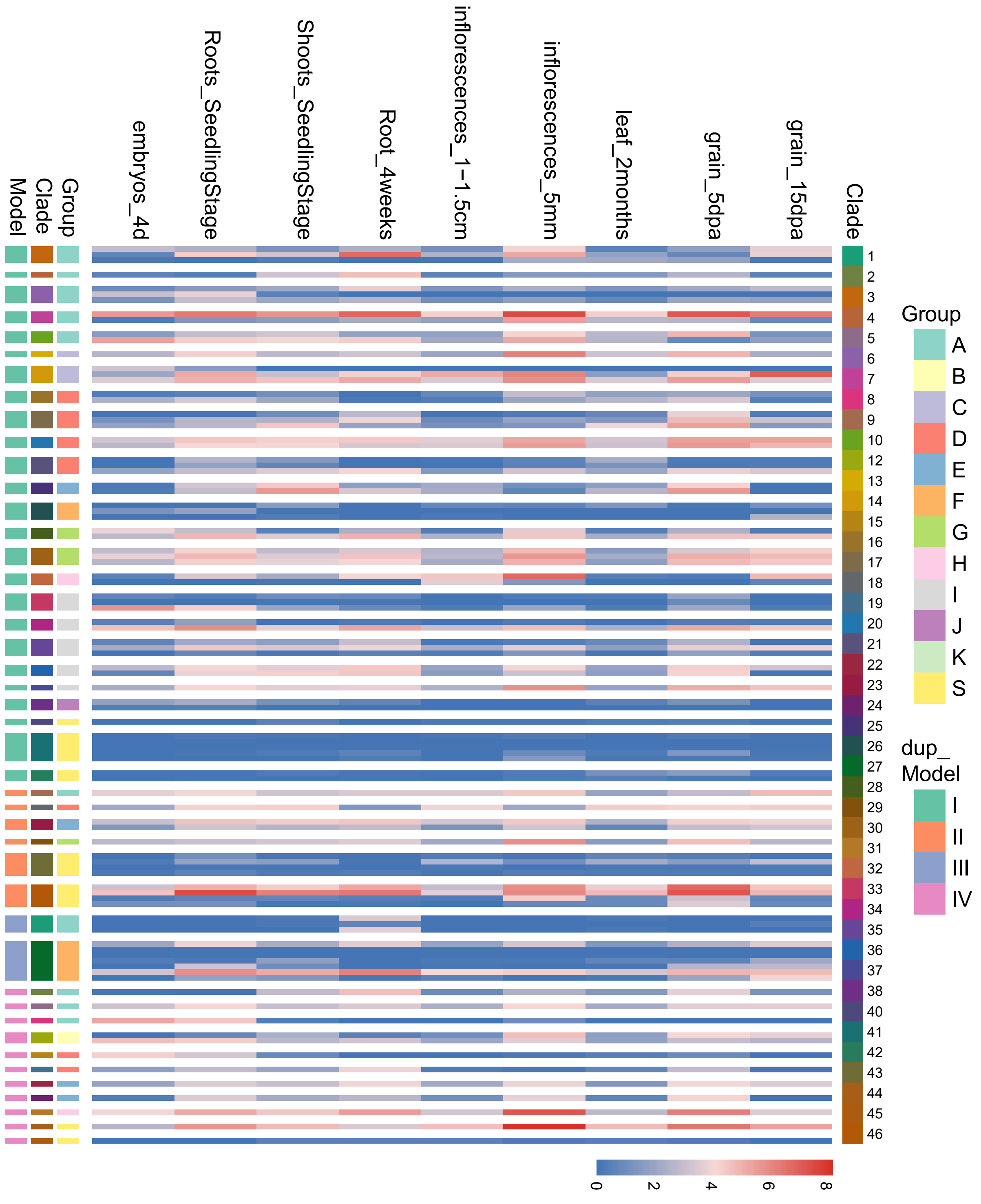

Figure S3. Robustness analysis of expression classification under different TPM thresholds. Venn diagrams showing the overlap between expression categories and stress-responsive gene sets under three TPM thresholds: (A) TPM ≥ 0.5, (B) TPM ≥ 1, and (C) TPM ≥ 2. The upper panels show overlaps between broadly expressed genes and genes responsive to at least three stress conditions (≥3 stress responsive). The middle panels show overlaps between tissue-specific expressed genes and genes responsive to one stress condition (1 stress responsive). The lower panels show overlaps between non-expressed genes and non-responsive genes. Although the absolute numbers of genes in each category varied with TPM threshold, the overall expression patterns and major trends remained qualitatively consistent across thresholds, supporting the robustness of the classification scheme.

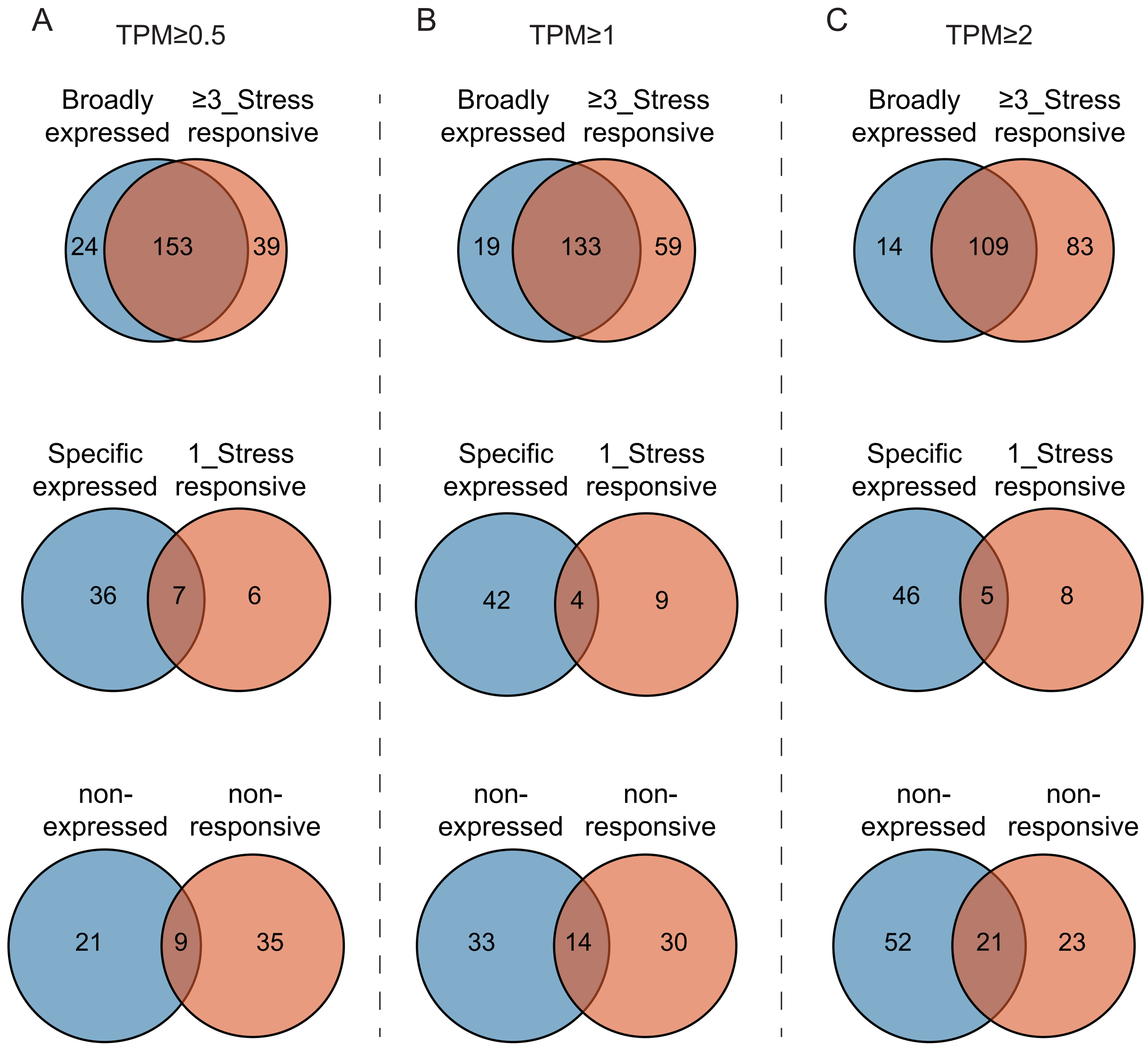

Supplement: Supplementary file 1 [file 3_Supplementary_Figure.pdf › 3_Supplementary_Figure.pdf]
